# Gibberellin pathway remodeling accompanies ethylene-driven sex reversal in *Cannabis sativa*

**DOI:** 10.3389/fpls.2026.1905836

**Published:** 2026-09-09

**Authors:** Julien Roy, Adrian S. Monthony, Davoud Torkamaneh

**Affiliations:** 1 Département de phytologie, Université Laval, Québec, QC, Canada; 2 Institut de Biologie Intégrative et des Systèmes (IBIS), Université Laval, QC, Canada; 3 Centre de recherche et d’innovation sur les végétaux (CRIV), Université Laval, QC, Canada; 4 Institut intelligence et données (IID), Université Laval, QC, Canada; 5 Institut sur la nutrition et les aliments fonctionnels (INAF), Université Laval, QC, Canada

**Keywords:** cannabis, ethylene, gibberellin, sex determination, sexual plasticity, transcriptomics

## Abstract

**Introduction:**

Sex expression in *Cannabis sativa* is determined by XX/XY sex chromosomes but remains plastic, with ethylene inhibition inducing male flowers on XX plants and ethylene release inducing female flowers on XY plants. Although ethylene is a central regulator of this process, the contribution of the gibberellin (GA) pathway to cannabis sex reversal remains poorly defined.

**Methods:**

We reconstructed the GA biosynthesis, regulation, and signaling pathway in *C. sativa* using orthology-based searches, and profiled GA-related gene expression during chemically induced sex reversal through transcriptomic analyses spanning the vegetative baseline, early post-treatment leaves, and developing flowers.

**Results:**

Orthology-based searches identified 50 putative *C. sativa* GA-related genes distributed across the genome, with 11 on the X chromosome, including six in the non-recombining region. Expression profiles were broadly similar between XX and XY plants at day 0, weakly perturbed at day 1, and strongly structured by floral phenotype at day 14. Early responses were limited to downregulation of *CsGA3ox1* in ethephon-treated XY plants and *CsGASA1* in STS-treated XX plants. By day 14, sex reversal was associated with differential expression of *CsGA1*, multiple *GA20ox* orthologs, *CsGID1B*, *CsSLY2*, and several *GASA* genes, indicating broad remodeling of GA-related transcription.

**Discussion:**

These results show that ethylene-pathway manipulation is associated with time- and phenotype-dependent changes in GA-related gene expression, pointing to a contribution of the GA pathway to cannabis sex reversal.

## Introduction

Sexual reproduction in angiosperms encompasses a remarkable diversity of breeding systems. Most flowering plant species produce bisexual (hermaphroditic) flowers bearing both male and female organs, a condition that facilitates self-pollination and reproductive assurance (Barrett, 2002; Renner, 2014). However, a minority of angiosperms have evolved unisexual flowers, giving rise to monoecious species, in which separate male and female flowers develop on the same individual, and dioecious species, in which male and female flowers are borne on distinct individuals (Charlesworth, 2002). Dioecy has evolved independently hundreds of times across the angiosperm phylogeny and occurs in approximately 5–6% of flowering plant species (Renner, 2014). In dioecious lineages, sex is often determined by sex chromosomes, which may range from homomorphic pairs with limited differentiation to highly heteromorphic systems resembling those of animals (Ming et al., 2011). The evolution of sex chromosomes in plants is thought to proceed through the progressive suppression of recombination around a sex-determining locus, leading to the accumulation of sex-linked genes and, eventually, morphological differentiation of the sex chromosome pair (Bergero and Charlesworth, 2009).

Despite the presence of genetic sex-determining mechanisms, sexual expression in many plant species is not rigidly fixed. In both dioecious and monoecious plants, the phenotypic sex of flowers can be modulated by environmental conditions, developmental stage, and phytohormone signaling, a phenomenon broadly referred to as sexual lability or sexual plasticity (Cossard and Pannell, 2021; Käfer et al., 2022). Sexual plasticity has been documented across diverse taxa, including *Carica papaya* (papaya), *Spinacia oleracea* (spinach), *Mercurialis annua*, and *Amborella trichopoda* (Anger et al., 2017; Cossard and Pannell, 2021; Lin et al., 2016). The triggers that shift phenotypic sex or floral sex ratios vary widely and include natural population variability, reproductive pressure, abiotic or biotic stress, and targeted molecular interventions with plant growth regulators (Dennis Thomas, 2004; J. Zhang et al., 2017). Among the phytohormones implicated in sex determination, ethylene and gibberellins (GAs) have emerged as central players, with evidence from multiple plant families linking their biosynthesis and signaling to the control of male versus female flower development (Chailakhyan and Timiriazev, 1979; Chandler, 2011; Diggle et al., 2011).

*Cannabis sativa* L. is a predominantly dioecious species with dimorphic male and female flowers on separate individuals (Bonini et al., 2018). The species has 10 chromosome pairs (2n = 20), comprising nine autosomal pairs and one sex-chromosome pair: males are typically XY with heteromorphic sex chromosomes, whereas females are XX with a homomorphic pair (Carey et al., 2026; Prentout et al., 2020). Pistillate flowers (female flowers; FF) comprise an ovary enclosed by two bracts bearing glandular trichomes and terminate in two elongated stigmas (Leme et al., 2020; Spitzer-Rimon et al., 2019). Staminate flowers (male flowers; MF) consist of a simple perianth and typically five stamens positioned opposite the sepals (Schilling et al., 2020). The glandular trichomes found in female inflorescences produce a diversity of cannabinoids with substantial pharmacological interest (Andre et al., 2016). In recent years, the cannabis genome has been sequenced and assembled (Grassa et al., 2021; Van Bakel et al., 2011), and interest in the genetic and molecular basis of sex determination has grown considerably (Adal et al., 2021; Chen et al., 2025; Monthony et al., 2026; Orozco et al., 2026; Prentout et al., 2020; Shi et al., 2025).

As observed in other dioecious plants, sex expression in *C. sativa* is not determined solely by sex chromosomes. Under specific environmental or experimental conditions, cannabis plants can display flowers that do not align with their chromosomal sex (XX/XY karyotype), a phenomenon recognized for decades (Chailakhyan and Timiriazev, 1979; Mohan Ram and Sett, 1982). Among the hormonal regulators implicated in this plasticity, ethylene emerged as a key modulator of feminization: inhibition of ethylene signaling using silver thiosulfate (STS) or silver nitrate reliably induces male flowers on XX plants (induced male flowers; IMF), whereas exogenous applications of ethylene or the ethylene-releasing compound ethephon induce feminization of XY plants (induced female flowers; IFF) (Flajšman et al., 2021; Garcia-de Heer et al., 2025; Monthony et al., 2026; Moon et al., 2020). Conversely, GAs have been recognized as modulators of sex expression since the pioneering work demonstrating that exogenous GA_3_ applications can induce male flowers on XX plants (Galoch, 1978; Ram and Jaiswal, 1972). These chemical interventions now form the basis of feminized seed production in the cannabis industry, yet the underlying molecular mechanisms governing induced sex change have remained poorly understood until recently.

Recent transcriptomic and multi-omics investigations have begun to uncover the molecular architecture of ethylene-mediated sexual plasticity in cannabis. A comprehensive study by Monthony et al. (2026) integrated over 130 RNA-seq libraries with ethylene pathway metabolite profiling and whole-genome sequencing across three XX and XY genotypes treated with STS and ethephon, respectively. This work demonstrated that ethylene-mediated sexual plasticity involves both systemic and local signaling components, where early transcriptional activation of ethylene biosynthesis and signaling genes occurred within 18 hours of treatment in leaves, prior to the emergence of flowers, while the new phenotypic sexual identity in developing flowers involved distinct sets of genes differentially regulated in each chromosomal sex. Other transcriptomic studies have similarly identified ethylene-related genes as candidates for sex determination (Adal et al., 2021), and network ontology analyses have further highlighted hormone-related gene expression changes associated with sexual plasticity (Orozco et al., 2026). Together, these findings have established ethylene as a primary hormonal driver of sexual plasticity in cannabis but also raised the question of whether other phytohormone pathways, particularly GAs, participate in this regulatory network.

GAs comprise a large family of tetracyclic diterpenoid phytohormones that regulate diverse aspects of plant growth and development, including stem elongation, flowering, fertility, and reproductive organ development (Bao et al., 2020; Pimenta Lange and Lange, 2006; Plackett et al., 2011; Wilson et al., 1992; Yamaguchi, 2008; Yu et al., 2004). Although more than one hundred gibberellins have been identified, only a small subset, primarily GA_1_ and GA_4_ in plants, are bioactive (Shani et al., 2024), with the remaining acting as precursors or inactive forms (Hedden and Thomas, 2012). *In planta* GA activity largely depends on the balance between GA production and deactivation (Yamaguchi, 2008). GA biosynthesis is a complex, multi-step process beginning in the plastid with a 20-carbon precursor, geranylgeranyl diphosphate (GGPP), which undergoes enzymatic transformation to form GA_12_, the common precursor for all GAs in plants (He et al., 2020; Shani et al., 2024). From GA_12_, two parallel branches are commonly described: a non-13-hydroxylated route yielding GA_4_ and a 13-hydroxylated route in which GA_12_ is hydroxylated by GA13-oxidase (GA13ox) to GA_53_ and ultimately converted to GA_1_. Following synthesis in the endoplasmic reticulum, both GA_12_ and GA_53_ are converted in the cytosol via GA20-oxidases (GA20ox), which generate C19 precursors (e.g., GA9/GA20), and GA3-oxidases (GA3ox), which convert these into the bioactive GAs GA_4_/GA_1_ (Shani et al., 2024). GA deactivation is classically mediated by GA2-oxidases (GA2ox), which can also remove precursors from the biosynthetic pool.

Perception and signaling of bioactive GAs occur primarily in the nucleus, where GA binds to the soluble receptor GA INSENSITIVE DWARF1 (GID1) (Griffiths et al., 2007; Hirano et al., 2008) and promotes formation of a complex that triggers DELLA protein degradation via SLEEPY1 (SLY1) or its homolog SNEEZY (SNE/SLY2), both F-box subunits of SCF E3 ubiquitin ligase complexes (Ariizumi et al., 2011; McGinnis et al., 2003). DELLA proteins, belonging to the GRAS family, function as master growth repressors that integrate multiple phytohormone signals; their GA-dependent degradation de-represses downstream developmental programs (Davière and Achard, 2013). DELLA activity is further modulated by SPINDLY (SPY) and SCARECROW-LIKE (SCL). When GA activity is low, DELLAs accumulate and activate feedback mechanisms that modulate transcriptional regulation of GA metabolic genes, including *GA20ox*, *GA3ox*, and *GA2ox*, in several systems (Fukazawa et al., 2014; Hedden and Thomas, 2012). Downstream of these core signaling events, GA-responsive genes are induced, including members of the GA-stimulated transcripts (*GASA/GAST1-like*) family, which encode small, secreted, cysteine-rich peptides that serve as integration nodes for multiple hormonal pathways (Aubert et al., 1998; Qu et al., 2016; Roxrud et al., 2007).

An additional feature of GA biology is its spatial organization. GA biosynthesis, deactivation, perception, and response need not occur in the same cells or tissues: expression of early and late biosynthetic enzymes can be spatially separated, requiring movement of pathway intermediates for local production of bioactive GA (Binenbaum et al., 2018; Olszewski et al., 2002). GAs and their precursors can also move between cells and organs, while localized biosynthesis, catabolism, and transport together generate tissue- and developmental-stage-specific GA distributions (Dayan, 2016; Rizza and Jones, 2019).

Beyond their roles in growth regulation, GAs serve as sex determinants across a range of plant lineages: in ferns and most eudicots, GAs generally promote male organ development, while in some monocots such as maize the effect is reversed (Gupta and Chakrabarty, 2013; Vandenbussche et al., 2007). In *Arabidopsis thaliana*, GA is essential for stamen filament elongation and anther development, and GA-deficient mutants display severe male sterility (Cheng et al., 2004; Plackett et al., 2011). Foundational work in the dioecious species *S. oleracea* implicated GA signaling in unisexual floral development: GA application promoted male-organ development in female plants, whereas inhibition of GA biosynthesis or proteasome activity induced female organs in males (West and Golenberg, 2018). Although bulk GA concentrations did not differ between the sexes, *SpGAI*, the only detected DELLA-family gene, showed higher inflorescence expression in females; its silencing promoted male-organ development. These findings identified sex-biased DELLA regulation as a component of the spinach feminizing pathway. Subsequent transcriptomic studies further linked GA-related regulation to spinach floral sex. Li et al. (2020) identified sex-biased co-expression networks in male and female flowers in which auxin- and GA-related genes were prominent. A *GAST1/GASA13* ortholog and a *GASA6* ortholog were the genes most closely co-expressed with the central hub genes of the female and male networks, respectively, identifying GASA-family genes as candidate components of sex-biased regulatory networks. Similarly, gene regulatory network analysis across female, male, and monoecious spinach flowers implicated GA and ABA signal-transduction pathways as major components of floral regulatory networks (Ma et al., 2024).

Functional evidence was subsequently provided by Zhang et al. (2024). In spinach, GA3 treatment induced functional masculinization of genetically female plants, and silencing *GIBBERELLIC ACID INSENSITIVE* (*SpGAI*), which encodes the single DELLA repressor in this system, produced a similar masculinized phenotype with viable pollen. Zhang et al. further showed that SpGAI interacts with the *KNOX* transcription factor SpSTM to repress the B-class floral identity gene *SpPI*, regulating female floral development. More recently, Wang et al. (2025) showed that GA treatment can also induce stamen carpelization in male spinach, accompanied by changes in GA, auxin, cytokinin, jasmonate, and ABA profiles. Their functional analyses implicated the anther-associated genes *SpAMS* and *SpPGIP* as activators of *SpPI*. Together, these studies show that GA can influence sex-associated floral development through DELLA-dependent regulation and broader hormone- and anther-developmental networks in spinach.

In *C. sativa*, a genome-wide association study identified a major sex-determination QTL (QTLSex_det1) containing a gene encoding a GAI-like DELLA protein (Petit et al., 2020), identifying a DELLA-related candidate but not establishing a conserved mechanism (Salentijn et al., 2019). Alter et al. (2024) showed that inflorescence development in female *C. sativa* is mediated by photoperiod and GA: short-day conditions trigger a decrease in bioactive GA4 at the shoot apex that is required for compact inflorescence formation, whereas exogenous GA3 prevents condensation. Transcriptomic analyses of sex-changed cannabis plants have also identified GA-related genes associated with sexual plasticity. In IMF on XX plants, a putative GA2ox gene is consistently downregulated, suggesting altered transcriptional regulation of GA deactivation in masculinized tissues (Toscani et al., 2026). *CsGASA4* was upregulated in both MF and STS-induced IMF relative to untreated FF (Adal et al., 2021), and a cytochrome P450 gene proposed to participate in GA biosynthesis was consistently upregulated in male tissues (Orozco et al., 2026).

Crucially, the GA and ethylene pathways do not operate in isolation. In model species, GA–ethylene crosstalk is mediated largely through DELLA proteins, which serve as integration hubs for both pathways. Ethylene signaling stabilizes DELLA proteins, thereby reducing GA-responsive growth and development (Achard et al., 2003, 2007). Activated ethylene signaling can also reduce levels of bioactive GAs by inhibiting GA biosynthesis enzymes such as GA20ox (Achard et al., 2007), and the ethylene-regulated transcription factor *EIN3* can physically interact with the *JAZ*-family repressors, which themselves interact with DELLAs (Colebrook et al., 2014; Davière and Achard, 2013). In certain developmental contexts, ethylene and GA can act either antagonistically or synergistically: ethylene inhibits root elongation by blocking GA-induced DELLA degradation, while in floral induction GA promotes flowering and ethylene typically delays it (Achard et al., 2007; Sun, 2008). These molecular interactions provide a framework for testing how changes in one pathway may be associated with responses in the other and why ethylene-modulating treatments in cannabis lead to coordinated shifts in both ethylene- and GA-related gene expression.

Despite well-documented phenotypic effects of manipulating GA and ethylene pathways in *C. sativa*, the transcriptional relationship between the pathways during sex reversal remains poorly characterized. Building on the ethylene-focused experimental framework of Monthony et al. (2026), this study reconstructs the canonical GA pathway in *C. sativa* and examines GA-related gene expression across treated and untreated plants at multiple time points. We test whether these transcript patterns are associated with ethylene-pathway manipulation, sampling time, and floral phenotype. This descriptive framework identifies candidates for future mechanistic study of GA in cannabis sex reversal.

## Materials and methods

### Identification of orthologs of GA-related genes in *C. sativa*

A comprehensive literature review was undertaken to compile a list of GA-related genes in the canonical GA biosynthesis, regulation, and signaling pathways (Castro-Camba et al., 2022; Davière and Achard, 2013; Hedden, 2020; Hedden and Thomas, 2012; Sun and Gubler, 2004; Yamaguchi, 2008). Based on the model species *A. thaliana*, orthologs of GA-related genes were identified in *C. sativa* using OrthoFinder (Emms and Kelly, 2015) and BLASTP searches (Camacho et al., 2009) for unmatched genes of interest. For *C. sativa*, the reference genome ASM2916894v1 (cultivar ‘Pink Pepper’; RefSeq accession GCF_029168945.1) was used, alongside the fully phased haploid X and Y genomes from genotype 284 ‘Otto II’ (Carey et al., 2026) to improve orthogroup identification on sex chromosomes. The TAIR10.1 (RefSeq accession GCF_000001735.4) served as the *A. thaliana* reference. Nucleotide and protein sequences were retrieved from NCBI. The chromosome map was generated using chromoMap (v4.1.1) (Anand and Rodriguez Lopez, 2022) in RStudio (2025.5.1.513) running R (v4.5.0).

### Plant selection, flowering and sex plasticity induction

The full vegetative plant propagation, growth conditions, chromosomal sex determination, flowering induction and sex plasticity treatments are outlined in detail by Monthony et al. (2026). Briefly, 8 vegetative clones of a known sex (XX or XY) from three *C. sativa* genotypes (La Rosca; *LR*, Panama Pupil V4; *PP*, Deadly Kernel; *DK*) were rooted for 2 weeks, for a total of 48 plants (two plants were lost, for a final 46 plants). Rooted clones were transferred to 10 cm square pots filled with Pro-Mix BX substrate (Pro-Mix, Canada), and grown under controlled conditions (Conviron, Canada) for 10 days to allow root establishment, then transplanted into 4-liter round pots and grown for a further 12 days under an initial 18/6 photoperiod, before the photoperiod was shifted to 12/12 to induce flowering. Light intensity from LED lighting reached up to 750 µmol/m²/s, and substrate pH was maintained between 5.5 and 6.0. Relative humidity was maintained around 70% (day) and 65% (night) during vegetative growth and reduced to 60% (day) and 50% (night) at the flowering stage. Temperature was maintained at 27 °C (day) and 25 °C (night) during vegetative growth, and at 25 °C (day) and 23 °C (night) at the flowering stage.

Sex reversal in half of the XX plants was induced using a 3 mM silver thiosulfate (STS) solution with 0.1% Tween 20, applied as foliar spray to saturation (~50 mL/plant) weekly for three weeks starting at the photoperiod shift (day 0). XY plants received a single application of 500 mg/L ethephon (diluted from 40% stock) with 0.1% Tween 20 at day 0. Ethephon was sprayed to saturation (~50 mL/plant) in a single application administered at the onset of the first 12-hour dark period, immediately following the photoperiod shift, to prevent leaf burning. In both cases, untreated control plants were sprayed with distilled water containing 0.1% Tween 20 to ensure comparable conditions. Each group had 3 or 4 biological replicates in each genotype, for a total of 11 FF, 12 IMF, 12 IFF and 11 MF plants (n = 46).

### RNA sequencing and transcriptome analysis

RNA extraction, library preparation, sequencing, and read processing steps are described in detail in Monthony et al. (2026). Mature leaf tissues were harvested at day 0 and 1 (18 hours after treatment), and immature flowers were harvested at day 14, for a total of 138 samples (46 plants, 3 time points). These samples were flash-frozen, ground, and total RNA was extracted using the RNeasy Plant Mini Kit (Qiagen GmbH, Germany). RNA quality was assessed by NanoDrop and Bioanalyzer, and samples with high integrity were used for cDNA synthesis with the NEBNext^®^ Ultra™ II Directional RNA Library Prep Kit (New England Biolabs, Ipswich, MA, USA). Sequencing was performed at the *Institut de Biologie Intégrative et des Systèmes* (IBIS; Université Laval) on the Element AVITI platform, producing 150 bp paired-end reads (~30 million reads/sample). Reads were quality-checked with FastQC and trimmed using Trimmomatic. Contaminant sequences (e.g., animal, fungal RNA) were removed by BLAST filtering using a custom Trimmomatic pipeline. Cleaned reads were aligned to the *C. sativa* ‘Pink Pepper’ genome using STAR (v2.7.11b), gene-level quantification was performed with HTSeq-count (v2.0.2) and mapping quality was verified with Qualimap (v.2.2.1; Okonechnikov et al., 2016). RNA-seq data generated for this study are deposited in the NCBI Sequence Read Archive (SRA) under BioProject accession PRJNA1404156.

### Differential expression analysis and gene expression visualizations

Differentially expressed GA-related genes were identified using DESeq2 (v1.42.1; Love et al., 2014). Low-count genes were filtered using HTSFilter (v1.42.0) (Rau et al., 2013) and normalized using variance stabilizing transformation (VST). For day 0 and day 1 samples, correction was applied as described in Roy et al. (2026). The log2FC values were shrunk using the *ashr* method to improve estimates for low-expression genes. Differentially expressed genes were defined using an adjusted P-value ≤ 0.05 and |log2FC| > 1. Candidate GA-related genes emphasized in the model were further required to meet both thresholds for the corresponding treatment contrast in all three genotypes. Results were visualized using bar plots of log2FC and boxplots of normalized counts for group comparisons using the R packages ggplot2 (v3.4.1; Wickham, 2016). The VST-normalized, batch-corrected counts of genes of interest were represented through principal components analysis (PCA) plots using ggplot2 (v3.5.1) (Wickham, 2016). All analyses were performed in RStudio (2025.5.1.513) running R (v4.5.0).

## Results

### Primary GA-related genes are present in the *C. sativa* genome

**Figure 1:**
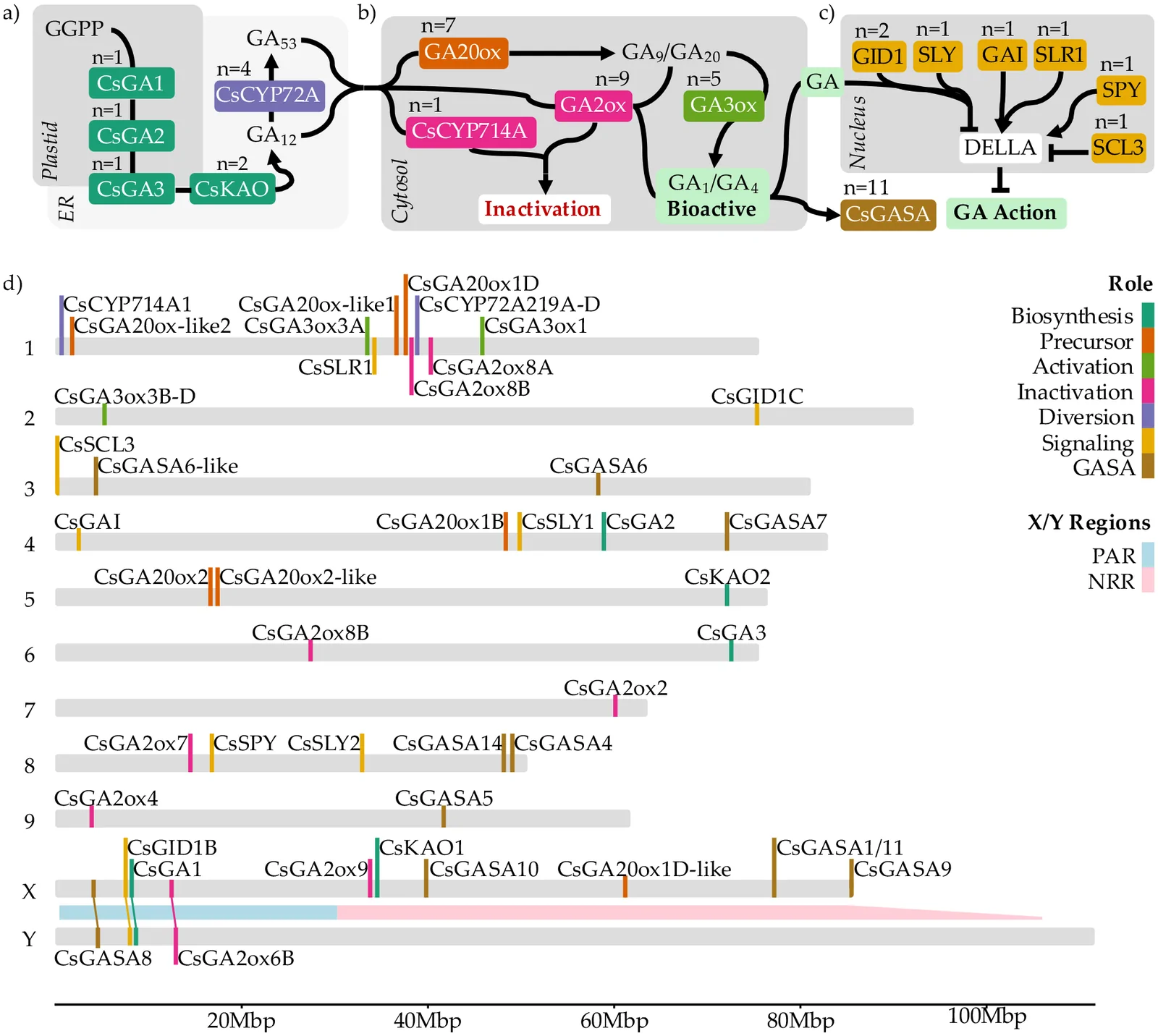
Proposed GA biosynthesis and signaling pathway based on orthology with *A. thaliana*, showing the number of orthologs found for each step and genomic localization of putative GA-related genes in *C. sativa*. **(a)** GA early biosynthesis, converting geranylgeranyl diphosphate (GGPP) to GA12 or GA53 occurring in the plastid and endoplasmic reticulum (ER). **(b)** In the cytosol, GA12/53 are converted to the bioactive GAs GA1 and GA4. **(c)** GA signaling in the nucleus, where bioactive GA binds the receptor GID1, leading to DELLA degradation and de-repression of GA-responsive genes. GA action induces GA-stimulated (GASA) genes. **(d)** Chromosomal positions of GA-related genes across autosomes and sex chromosomes X/Y, color-coded by functional role. Autosomal gene positions are based on the ‘Pink Pepper’ reference genome; pseudoautosomal region (PAR) and non-recombining regions (NRR) on the X and Y chromosomes follow Carey et al. (2026).

Orthology-based searches identified 50 putative GA-related genes, of which 46 were expressed above the filtering threshold in the transcriptomic dataset and are summarized in **Supplementary Table 1**. The gene collection is publicly available on the National Center for Biotechnology Information website (NCBI) (Bethesda, MD) (https://www.ncbi.nlm.nih.gov/sites/myncbi/julien.roy.2/collections/63974920/public/). This gene set covers the major enzymatic, regulatory, and signaling components of the canonical GA pathway. Putative orthologs were identified for the early biosynthetic steps leading to GA_12_, including CPS, KS, KO, corresponding to *CsGA1*, *CsGA2*, *CsGA3*, and two KAO paralogs (*CsKAO1*, *CsKAO2*; **Figure 1a**). Downstream of GA_12_, multiple paralogs were identified in the three oxidase families controlling GA activation and inactivation, namely GA20ox, GA3ox, and GA2ox (**Figure 1b**). The GA3ox group included *CsGA3ox1* and the *CsGA3ox3* series, with four closely related paralogs, *CsGA3ox3A*–D, including three neighboring genes. Additional GA-modifying enzymes were also identified, including one putative CYP714A gene (*CsCYP714A1*) and four putative GA13ox-like genes (*CsCYP72A219A*–D), supporting a reconstructed pathway that includes a GA13ox step connecting GA_12_ to GA_53_.

Core GA signaling components were also mapped, including genes encoding two putative GA receptors (*CsGID1B*, *CsGID1C*), DELLA-related genes (*CsGAI*, *CsSLR1*) and the signaling regulator *CsSCL3*, as well as the F-box genes *CsSLY1* and *CsSLY2*, which are involved in DELLA degradation (**Figure 1c**). The dataset also included the negative regulator *CsSPY* and multiple *GASA* genes representing GA-responsive outputs.

Genomic localization of these GA-related genes revealed a widespread distribution across the *C. sativa* genome (**Figure 1d**). The X chromosome harbored the highest number of GA-related genes (11), five of which were in the pseudoautosomal region (PAR) of the sex chromosomes. No genes were detected in the non-recombining region (NRR) of the Y chromosome, whereas six were located within the X chromosome NRR.

### Transcriptome results

Following filtration of low-count genes, 34 of the 50 GA-related genes remained in leaf samples (day 0 and day 1); full per-gene expression and differential expression statistics for this candidate gene set are reported in **Supplementary Table 2**. Genome-wide, DESeq2 identified 1133 significantly differentially expressed genes (DEGs; padj < 0.05, |log2FC| > 1) in the IMF vs. FF contrast, 3345 in the MF vs. FF contrast, and 917 in the MF vs. IFF contrast at day 1, with upregulated and downregulated gene counts for each comparison given in **Supplementary Table 4**. Volcano plots for each contrast are shown in **Supplementary Figure 1**, and a heatmap of the 4632 significantly differentially expressed genes across the three day 1 contrasts is provided in **Supplementary Figure 2**. Gene Ontology (GO) and KEGG pathway enrichment analyses of these leaf-tissue DEGs are provided in **Supplementary Figures S3–S8**.

**Figure 2:**
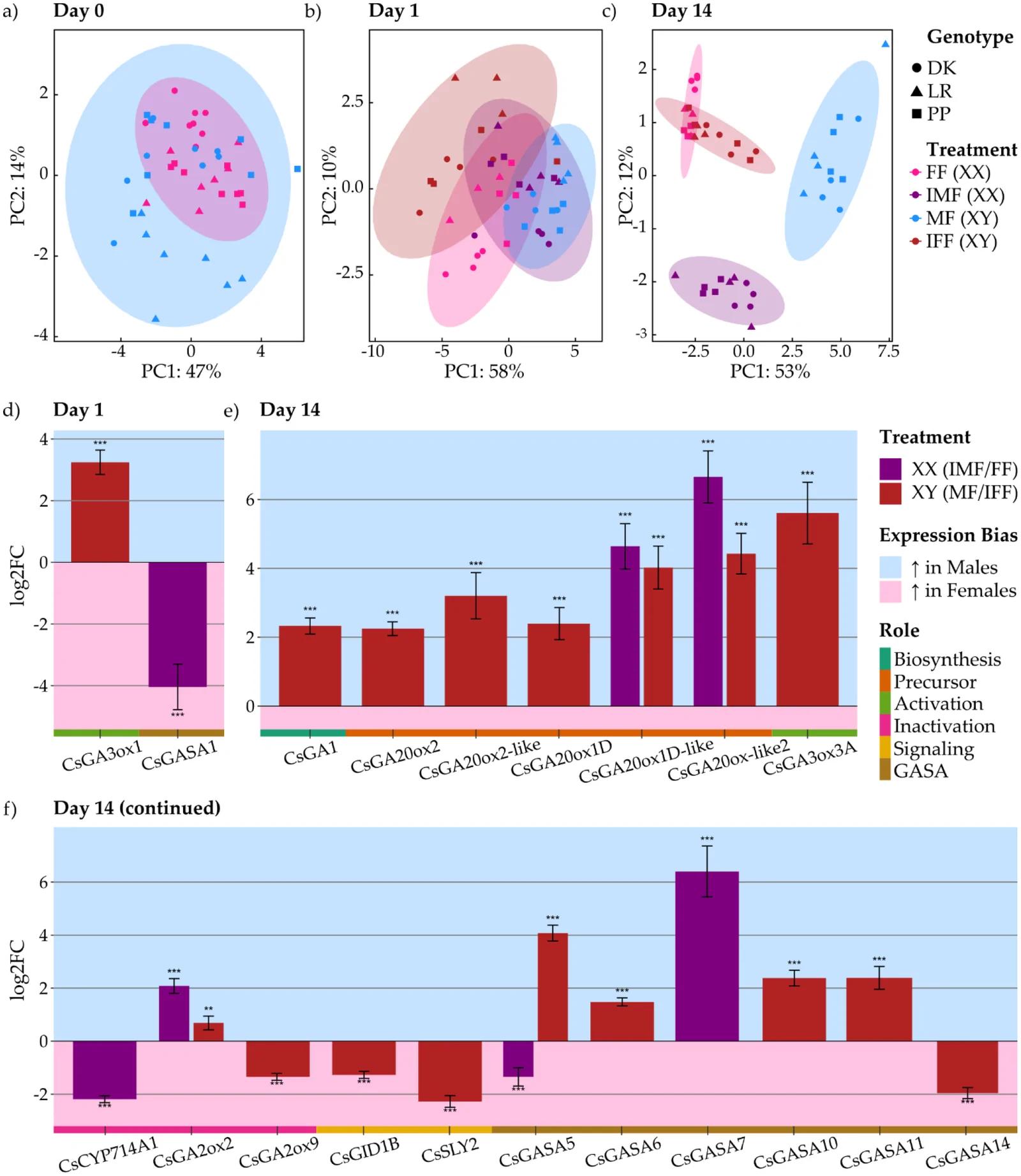
GA-related gene expression profiles across treatments and time points. **(a–c)** Principal component analysis (PCA) of batch-corrected variance-stabilizing transformed (VST) counts of GA-related genes. **(a)** Day 0 leaf samples (n = 34 genes); **(b)** Day 1 leaf samples (n = 34 genes); **(c)** Day 14 immature flower samples (n = 45 genes). Each point represents an individual sample; ellipses show 95% confidence intervals for each treatment group. Colors indicate treatment groups and symbols represent genotypes (DK = Deadly Kernel; LR = La Rosca; PP = Panama Pupil V4). **(d)** Differential expression of GA-related genes at day 1 following ethylene pathway manipulation, grouped by functional role. Bars represent log2FC values relative to untreated controls for silver thiosulfate (STS) treatment on XX plants (IMF vs. FF, purple) and ethephon treatment on XY plants (MF vs. IFF, red). **(e, f)** Differential expression of GA-related genes at day 14 for the STS (XX) and ethephon (XY) contrasts, grouped by functional role; panel f continues panel **(e)** Black error bars indicate the DESeq2-reported lfcSE for the shrunken log2FC estimates. Genes with adjusted P-value ≤ 0.05 in all genotypes and |log2FC| >= 1 are displayed. Asterisks denote significance levels (*padj < 0.05; **padj < 0.01; ***padj < 0.001).

Principal component analysis (PCA) of GA-related gene expression revealed time-dependent structuring of samples according to phenotypic sex (**Figures 2a–c**). Baseline (day 0) analysis of expression profiles shows broad similarity during vegetative growth (**Figure 2a**), with PC1 and PC2 explaining 47% and 14% of the variance, respectively. Immediately following treatment and photoperiod change (day 1), expression profiles show perturbation, but do not cluster by chromosomal sex (XX vs. XY) or by sexual phenotype class, as indicated by the overlap of the 95% ellipses (**Figure 2b**), with PC1 and PC2 explaining 58% and 10% of the variance, respectively.

At day 14, 45 of the 50 GA-related genes were expressed in immature flower samples, with full per-gene differential expression results provided in **Supplementary Table 3**. Genome-wide, 3245 genes were significantly differentially expressed in the IMF vs. FF contrast, 7642 in the MF vs. FF contrast, and 5631 in the MF vs. IFF contrast at day 14, with upregulated and downregulated gene counts for each comparison given in **Supplementary Table 4**. Volcano plots for each contrast are shown in **Supplementary Figure 9**, and a heatmap of the 8603 significantly differentially expressed genes across the three day 14 contrasts is provided in **Supplementary Figure 10**. GO and KEGG enrichment analyses of these floral-tissue DEGs are shown in **Supplementary Figures S11–S16**.

At this stage, the PCA shows a more structured pattern of clustering (**Figure 2c**). FF (XX) and IFF (XY) samples overlap despite their different sex chromosome karyotypes, indicating clustering by sexual phenotype rather than genotype, with PC1 and PC2 explaining 53% and 12% of the variance, respectively. In contrast, phenotypic male samples form distinct groups: IMF (XX) does not overlap with MF (XY). Moreover, IMF (XX) samples are clearly separated from their untreated counterparts (FF), indicating a shift associated with ethylene inhibition and the acquisition of a male phenotype.

Only two GA-related genes responded in leaves within 18 hours of sex plasticity induction (Day 1). In XY plants, *CsGA3ox1* was significantly downregulated (log2FC = -3.24, adjusted P-value = 1.26e-15) following ethephon treatment compared to control (**Figure 2d**). In XX plants treated with STS, *CsGASA1* was significantly downregulated (log2FC = -4.04, adjusted P-value = 2.18e-7) relative to untreated XX controls (**Figure 2d**).

At day 14, several GA-related genes were differentially expressed across treatment groups (**Figures 2e, f**). In the ethephon contrast (MF vs. IFF; XY), *CsGA1* showed significantly higher expression in untreated immature flowers (log2FC = 2.33, adjusted P-value = 1.98e-23). Multiple GA20-oxidase paralogs (*CsGA20ox2*, *CsGA20ox2-like*, *CsGA20ox1D*, *CsGA20ox1D-like* and *CsGA20ox-like2*) were also significantly expressed at higher levels in untreated samples, indicating higher expression in phenotypic male flowers, with log2FC between 2.25 and 4.43. A GA3-oxidase gene showed strong downregulation following treatment, with higher expression in control males (log2FC = 5.61, adjusted P-value = 3.19e-11). In contrast, expression of *CsSLY2* was significantly lower in untreated males (log2FC = -2.27, adjusted P-value = 8.17e-25). Members of the GASA gene family displayed strong responses, with many paralogs strongly downregulated by treatment (*CsGASA5*, *CsGASA6*, *CsGASA10*, *CsGASA11*), while *CsGASA14* showed lower counts in control plants (log2FC = -1.96, adjusted P-value = 3.47e-21).

In the STS contrast (IMF vs. FF; XX), four genes were significantly differentially expressed at day 14 (**Figures 2e, f**), including *CsGA20ox1D-like* (log2FC = 4.64, adjusted *P*-value = 2.25e-12) and *CsGA20ox-like2* (log2FC = 6.66, adjusted *P*-value = 6.00e-18), which showed the same phenotype-associated pattern observed in the ethephon contrast, with higher expression in phenotypic male flowers. *CsCYP714A1* was significantly downregulated by treatment (log2FC = -2.19, adjusted P-value = 9.84e-63) relative to untreated XX controls. Finally, *CsGASA7* was significantly upregulated in IMF (log2FC = 6.40, adjusted P-value = 4.75e-11).

## Discussion

### GA-related transcriptional responses during chemically induced sex reversal

A feature of phytohormone signaling is a plant’s ability to exert temporal control over biological outcomes in response to internal and external stimuli. This study reconstructs a set of 50 putative gibberellin-related genes in *C. sativa* and shows that their expression changes during ethylene-modulated floral sex reversal. The principal finding is temporal: GA-related transcription was broadly similar between XX and XY plants at the vegetative baseline, only two GA-related genes responded in leaves within 18 h of treatment, and a substantially broader set of GA-related genes was differentially expressed in developing flowers at day 14.

At day 1, only two GA-related genes showed treatment-associated expression changes in leaf tissue. In ethephon-treated XY plants (IFF), *CsGA3ox1*, the sole *GA3ox* detected in leaves, was strongly downregulated. Because GA3ox enzymes catalyze the final activation step in GA biosynthesis, this result is consistent with altered expression of a GA-biosynthetic component, although GA abundance and enzyme activity were not measured. In STS-treated XX plants (IMF), *CsGASA1*, a putative downstream GA-responsive gene, was downregulated. Thus, the two treatments were associated with changes in different functional components of the GA-related gene network: a biosynthetic gene following ethephon treatment and a putative downstream response gene following STS treatment. These differences may reflect the treatments’ distinct modes or schedules of action, although the present data cannot distinguish among these possibilities. The limited number of responsive genes contrasts with the broader early ethylene-related response reported by Monthony et al. (2026), suggesting that GA-related transcriptional responses in leaves were limited at 18 hours and may occur later or primarily in developing floral tissue.

By day 14, GA-related transcriptional differences were more extensive and were associated with floral phenotype. In XY plants, untreated MF had higher expression of *CsGA1*, several *GA20ox* orthologs, and *CsGA3ox3A* than ethephon-treated IFF. In XX plants, two *GA20ox* orthologs were also more highly expressed in STS-IMF than in untreated FF. Because GA20ox enzymes contribute to the formation of GA precursors, the repeated association of *GA20ox* expression with phenotypically male flowers across both treatments is consistent with altered regulation of GA biosynthesis during the acquisition of male versus female phenotypic sex, particularly given that local conversion by GA20ox and GA3ox enzymes can contribute to bioactive GA pools in recipient tissues from mobile precursors such as GA_12_, a major long-distance mobile GA signal in *A. thaliana* (Binenbaum et al., 2018; Dayan, 2016).

Coordinated changes across multiple GA20ox orthologs were found in developing flowers, all of which showed higher expression in phenotypic male flowers than in phenotypic female flowers. This pattern is consistent with phenotype-associated regulation of GA biosynthetic genes. It cannot, however, be interpreted as evidence of elevated GA flux because GA abundance and enzyme activity were not measured, and GA20ox transcription can be subject to feedback regulation.

Notably, the genomic localization of *CsGA20ox1D-like* on chromosome X (position ~61.4 Mb), places it within a region that has been proposed to be subject to sex-chromosome-specific regulatory dynamics (Toscani et al., 2026).

The lower expression of *CsGA3ox3A* in IFF (XY) immature flowers than in untreated phenotypic males indicates treatment-associated transcriptional regulation of a GA-biosynthetic gene; it does not establish lower GA production. This ortholog shows comparatively low overall expression in our dataset relative to the predominantly expressed *CsGA3ox1*, and its expression is effectively restricted to XY plants under control conditions, with ethephon-treated XY plants showing expression reduced to near zero. Although *CsGA3ox1*, significantly downregulated in leaf tissue of IFF plants, did not meet the log2FC threshold, its expression in immature flowers was significantly higher in IMF (XX) than in control females (FF) (log2FC = 0.49, adjusted P-value = 0.006).

The expression of two distinct *CsGA2ox* orthologs in opposing sex-reversal contexts, one in IMF (XX) and another in IFF (XY), indicates treatment- and paralog-specific transcriptional responses. GA2ox enzymes deactivate bioactive GAs and their immediate precursors (Hedden and Thomas, 2012; Yamaguchi, 2008), but these transcript differences do not by themselves demonstrate altered GA pools or paralog-specific effects on GA homeostasis. In other species, GA2ox family members can differ in tissue specificity, substrate preference, and developmental role (Rieu et al., 2008; Shani et al., 2024). Whether the *C. sativa* GA2ox paralogs identified here are similarly functionally diversified remains an open question for future work.

*CsGA2ox9*, which was upregulated in IFF (XY), maps to the non-recombining region of the X chromosome. Its position and phenotype-associated expression are descriptive observations; they do not establish sex-biased regulation, shared control with nearby loci, or a role in sex determination. Paralog-specific functional analyses and direct GA measurements will be required to determine whether *CsGA2ox9* and *CsGA2ox2* differ in substrate preference, tissue specificity, or effects on GA homeostasis.

The lower expression of *CsGA1* and multiple GA20ox orthologs in IFF than in MF indicates coordinated transcriptional differences across several GA-biosynthetic steps. This pattern is consistent with ethylene-associated regulation reported in *A. thaliana* (Achard et al., 2007), but the present data do not demonstrate reduced GA abundance, suppression of pathway flux, or dismantling of a male floral GA program.

GA-related transcription may also covary with developmental differences between male and female inflorescences. Male plants typically show greater internode elongation, whereas female inflorescences are more compact, and Alter et al. (2024) linked shoot-apex GA4 dynamics to inflorescence condensation in female *C. sativa*. Accordingly, some phenotype-associated expression differences observed here may reflect inflorescence architecture or developmental stage rather than floral sex per se.

### GA signaling adjustments: *CsCYP714A1*, *CsGID1B* and *CsSLY2*

Beyond biosynthesis, our results revealed expression changes in genes annotated as GA modifiers or signaling components. *CsCYP714A1* was significantly downregulated in IMF relative to untreated female controls at day 14. In *A. thaliana*, CYP714A1 encodes a cytochrome P450 monooxygenase that diverts GA12, the common precursor of plant GAs, toward inactive forms; its overexpression produces severe GA-deficient dwarfism (Nomura et al., 2013; Y. Zhang et al., 2011). The *CsCYP714A1* expression change is consistent with altered transcription of a GA-inactivation component, but it does not demonstrate preservation of GA12, increased pathway flux, or higher bioactive GA abundance.

*CsGID1B* and *CsSLY2* were more highly expressed in IFF than in untreated MF at day 14. In model species, GID1 receptors mediate GA perception and SLY-family F-box proteins participate in DELLA degradation (Ariizumi et al., 2011; Griffiths et al., 2007; McGinnis et al., 2003). The observed transcript changes could therefore reflect altered receptor or F-box regulation, homeostatic feedback, or differences in floral developmental state. Because GA abundance, receptor activity, and DELLA protein abundance were not measured, these alternatives cannot be distinguished, and selective DELLA degradation cannot be inferred from the present data.

### GASA gene family members as downstream integrators of hormonal crosstalk

The GASA (Gibberellic Acid Stimulated Arabidopsis) gene family was among the most transcriptionally dynamic components examined during sex reversal. *CsGASA5*, *CsGASA6*, *CsGASA10*, and *CsGASA11* were more highly expressed in MF than in IFF, whereas *CsGASA14* showed the opposite pattern; *CsGASA7* was strongly upregulated in IMF relative to FF. GASA proteins are small, secreted, cysteine-rich peptides with roles in reproductive development and responses to several hormones (Aubert et al., 1998; Qu et al., 2016; Roxrud et al., 2007). Because GASA genes can respond to GA, brassinosteroids, auxin, abscisic acid, jasmonic acid, and salicylic acid, their expression cannot be treated as a GA-specific functional readout. Here, they are best interpreted as candidate markers of the broader hormonal and developmental state associated with floral phenotype.

Several GASA genes are located on the X chromosome (**Figure 1D**): *CsGASA1*, *CsGASA8*, *CsGASA9*, *CsGASA10*, and *CsGASA11*. *CsGASA8* is in the pseudoautosomal region, whereas the other four are in the non-recombining region; *CsGASA1* and *CsGASA11* are physically close to the monoecy locus reported by Carey et al. (2026). These positions are descriptive. The present expression data do not test shared regulation among these genes, regulatory effects of proximity to the monoecy locus, or a role for this chromosomal region in GA-ethylene crosstalk or sex determination.

### Evidence for GA–ethylene crosstalk during sexual plasticity

Work in *A. thaliana* and *S. oleracea* provides plausible mechanisms through which ethylene and GA pathways can interact, including DELLA-dependent feedback and regulation of floral identity genes (Achard et al., 2003, 2007; West and Golenberg, 2018; Zhang et al., 2024). The GA-related transcript shifts observed in *C. sativa* are consistent with crosstalk reported in those species. However, cross-species similarity does not establish that DELLA stabilization, GID1B feedback, selective SLY2 activity, or GA-dependent control drives cannabis sex reversal; the causal mechanism remains unresolved.

### Limitations and future directions

Taken together, the directly supported conclusion is that ethylene-pathway perturbation is associated with a limited early response in leaf tissue and broader, phenotype-associated remodeling of GA-related transcription in developing flowers. These patterns identify candidate biosynthetic, signaling, and response genes, but they do not establish the direction of crosstalk or a causal role for GA in sex reversal.

The interpretation of GA-related gene transcriptional dynamics warrants consideration of the spatial dimension of GA. Consequently, expression of a GA-related gene at one sampling site cannot by itself identify the site of GA accumulation, perception, or developmental action. This distinction is especially relevant here because early transcriptional responses were measured in leaves, whereas later responses were measured in developing flowers. Whether GA or GA precursors produced in vegetative tissues contribute to GA dynamics in developing cannabis flowers remains unknown. Furthermore, the well-documented feedback regulation of GA biosynthesis genes by DELLA accumulation means that elevated *GA3ox* or *GA20ox* transcript levels can reflect low bioactive GA instead of high biosynthetic output (Hedden and Thomas, 2012).

Future work should combine time- and tissue-resolved measurements of endogenous GA metabolites, DELLA protein abundance, enzyme activity, and GA-responsive outputs with perturbation of specific candidate genes. Such experiments could test whether the transcript patterns reflect GA feedback, broader floral development, or a causal contribution to sex reversal. Functional validation is now warranted, while recognizing that stable cannabis transformation and regeneration remain difficult and are not yet routine.

## Conclusion

This study reconstructs the *C. sativa* GA pathway and shows that ethylene-pathway manipulation is associated with time- and phenotype-dependent changes in GA-related gene expression during sex reversal. The results identify candidate biosynthetic, signaling, and response genes for future testing but do not establish changes in endogenous GA, DELLA abundance, or a causal role for GA in sex reversal.

## Data Availability

Publicly available datasets were analyzed in this study. This data can be found here: BioProject accession PRJNA1404156.
