# Supplementary material for: Gibberellin pathway remodeling accompanies ethylene-driven sex reversal in Cannabis sativa
Source: Front Plant Sci. 2026 Sep 9;17:1905836. doi: 10.3389/fpls.2026.1905836 (PMC13597699; doi:10.3389/fpls.2026.1905836)
Supplement: Supplementary file 1 [file DataSheet1.pdf]

Supplementary Table and Figures

Supplementary Table S1. Candidate gibberellin-related genes identified in *Cannabis sativa*. List of *C. sativa* genes involved in GA biosynthesis, deactivation, signaling, and GASA-domain-containing genes, identified based on sequence homology to *Arabidopsis thaliana* GA pathway genes. For each gene, the NCBI Gene ID (LOCID), assigned gene name, chromosomal location ("X/Y" indicates presence on both sex chromosomes; "Un" indicates unplaced scaffold), the corresponding *A. thaliana* ortholog(s), and functional category are provided. This gene list was used as the basis for GA-pathway-related genes in the differential expression analyses.

**LOCID**	**Name**	**Chrom.**	***A. thaliana* ortholog**	**Function**
LOC115703738	CsGA1	X/Y	AT4G02780	Biosynthesis
LOC115712980	CsGA2	4	AT1G79460	Biosynthesis
LOC115695692	CsGA3	6	AT5G25900	Biosynthesis
LOC115710887	CsKAO1	X	AT1G05160, AT2G32440	Biosynthesis
LOC115715978	CsKAO2	5	AT1G05160, AT2G32440	Biosynthesis
LOC115715725	CsGA20ox2	5	AT5G51810, AT4G25420	Precursor
LOC115716895	CsGA20ox2-like	5	AT5G51810	Precursor
LOC115704439	CsGA20ox1D	1	AT4G25420	Precursor
LOC115712114	CsGA20ox1B	4	AT5G51810	Precursor
LOC115694716	CsGA20ox1D-like	X	AT5G51810	Precursor
LOC115704916	CsGA20ox-like1	1	AT4G23340	Precursor
LOC115703660	CsGA20ox-like2	1	AT4G23340	Precursor
LOC115705300	CsGA3ox1	1	AT1G15550, AT1G80340	Activation
LOC115704454	CsGA3ox3A	1	AT4G21690	Activation
LOC115720796	CsGA3ox3B	2	AT4G21690	Activation
LOC133034677	CsGA3ox3C	2	AT4G21690	Activation
LOC115720809	CsGA3ox3D	2	AT4G21690	Activation
LOC115708157	CsCYP72A219A	1	AT3G14630	Modification
LOC115706712	CsCYP72A219B	1	AT3G14630	Modification
LOC115707772	CsCYP72A219C	1	AT3G14630	Modification
LOC115706721	CsCYP72A219D	1	AT3G14630	Modification
LOC115706085	CsCYP714A1	1	AT5G24910	Inactivation
LOC115706670	CsGA2ox1	1	AT1G78440	Inactivation
LOC115696581	CsGA2ox2	7	AT1G30040	Inactivation
LOC115723423	CsGA2ox4	9	AT1G47990	Inactivation
LOC115704443	CsGA2ox6A	Un	AT1G02400	Inactivation
LOC133032535	CsGA2ox6B	X/Y	AT1G02400	Inactivation
LOC115701137	CsGA2ox7	8	AT1G50960	Inactivation
LOC115704748	CsGA2ox8A	1	AT4G21200	Inactivation
LOC115695970	CsGA2ox8B	6	AT1G50960	Inactivation
LOC115710658	CsGA2ox9	X	AT5G58660, AT3G47190	Inactivation
LOC115712712	CsGAI	4	AT1G14920	Signaling
LOC115706185	CsSLR1	1	AT1G14920	Signaling
LOC115711337	CsSCL3	3	AT1G50420	Signaling
LOC115702835	CsGID1B	X/Y	AT3G63010	Signaling

LOCID	Name	Chrom.	*A. thaliana* ortholog	Function
LOC115719219	CsGID1C	2	AT5G27320, AT3G05120	Signaling
LOC115712738	CsSLY1	4	AT4G24210	Signaling
LOC115698964	CsSLY2	8	AT5G48170	Signaling
LOC115701130	CsSPY	8	AT3G11540	Signaling
LOC115698303	CsGASA1	X	AT1G75750, AT2G18420	GASA
LOC115701397	CsGASA4	8	AT5G15230	GASA
LOC115721929	CsGASA5	9	AT3G02885	GASA
LOC115708697	CsGASA6	3	AT1G74670	GASA
LOC115710205	CsGASA6-like	3	AT1G74670	GASA
LOC115715027	CsGASA7	4	AT2G14900	GASA
LOC115703025	CsGASA8	X/Y	AT2G39540	GASA
LOC115703246	CsGASA9	X	AT1G22690	GASA
LOC115714466	CsGASA10	X/Y	AT5G59845	GASA
LOC115698285	CsGASA11	X	AT2G18420	GASA
LOC133030008	CsGASA14	8	AT5G14920	GASA

Supplementary Table S2. Differential expression of gibberellin (GA) pathway genes at day 1 under IMF vs. FF and MF vs. IFF treatment contrasts. Log2 fold change (log2FC), standard error (lfcSE), and Benjamini-Hochberg adjusted p-value (padj) are shown for each gene from the candidate GA pathway gene list (Supplementary Table S1) that was detected in the leaf-tissue RNA-seq dataset at day 1. Values reflect the overall treatment effect for each contrast, estimated independently of cultivar. Log2FC values were obtained using ashr shrinkage estimation (lfcShrink, DESeq2). Genes are grouped by functional category. padj are indicated as: ns, not significant (padj ≥ 0.05); *, padj < 0.05; **, padj < 0.01; ***, padj < 0.001.

LOCID	Name	Function	IMF v. FF log2FC	IMF v. FF lfcSE	IMF v. FF padj	MF v. IFF log2FC	MF v. IFF lfcSE	MF v. IFF padj
LOC115703738	CsGA1	Biosynthesis	-0.09	0.20	ns	0.19	0.22	ns
LOC115712980	CsGA2	Biosynthesis	0.02	0.19	ns	0.34	0.32	ns
LOC115695692	CsGA3	Biosynthesis	-0.67	0.28	**	-0.08	0.17	ns
LOC115710887	CsKAO1	Biosynthesis	-0.10	0.24	ns	0.00	0.22	ns
LOC115715978	CsKAO2	Biosynthesis	0.08	0.35	ns	-0.34	0.65	ns
LOC115715725	CsGA20ox2	Precursor	0.36	0.33	ns	0.24	0.27	ns
LOC115716895	CsGA20ox2-like	Precursor	0.01	0.41	ns	0.02	0.38	ns
LOC115704439	CsGA20ox1D	Precursor	-0.02	0.27	ns	0.01	0.27	ns
LOC115712114	CsGA20ox1B	Precursor	-0.03	0.29	ns	0.11	0.35	ns
LOC115694716	CsGA20ox1D-like	Precursor	0.01	0.59	ns	0.00	0.45	ns
LOC115704916	CsGA20ox-like1	Precursor	0.04	0.41	ns	-0.06	0.40	ns
LOC115705300	CsGA3ox1	Activation	0.06	0.20	ns	3.24	0.39	***
LOC115704454	CsGA3ox3A	Activation	0.00	0.60	ns	0.00	0.45	ns
LOC115720796	CsGA3ox3B	Activation	0.01	0.60	ns	0.00	0.45	ns
LOC115720809	CsGA3ox3D	Activation	0.00	0.60	ns	0.00	0.45	ns
LOC115708157	CsCYP72A219A	Diversion	0.00	0.19	ns	-0.85	0.46	**
LOC115706712	CsCYP72A219B	Diversion	0.26	0.13	ns	-0.58	0.14	***
LOC115707772	CsCYP72A219C	Diversion	-0.01	0.13	ns	-0.19	0.16	ns
LOC115706721	CsCYP72A219D	Diversion	1.67	1.31	*	-0.19	0.41	ns
LOC115706085	CsCYP714A1	Inactivation	0.65	0.67	*	-0.24	0.38	ns

LOCID	Name	Function	IMF v. FF log2FC	IMF v. FF lfcSE	IMF v. FF padj	MF v. IFF log2FC	MF v. IFF lfcSE	MF v. IFF padj
LOC115706670	CsGA2ox1	Inactivation	0.02	0.26	ns	0.15	0.34	ns
LOC115696581	CsGA2ox2	Inactivation	0.24	0.27	ns	-0.27	0.27	ns
LOC115723423	CsGA2ox4	Inactivation	0.04	0.52	ns	0.01	0.41	ns
LOC115704443	CsGA2ox6A	Inactivation	0.05	0.47	ns	0.04	0.40	ns
LOC133032535	CsGA2ox6B	Inactivation	0.00	0.60	ns	0.00	0.45	ns
LOC115701137	CsGA2ox7	Inactivation	0.00	0.33	ns	-0.32	0.69	ns
LOC115704748	CsGA2ox8A	Inactivation	0.30	0.65	ns	0.16	0.42	ns
LOC115695970	CsGA2ox8B	Inactivation	0.04	0.21	ns	-0.14	0.25	ns
LOC115710658	CsGA2ox9	Inactivation	-0.12	0.19	ns	-0.04	0.18	ns
LOC115712712	CsGAI	Signaling	0.04	0.15	ns	0.18	0.18	ns
LOC115706185	CsSLR1	Signaling	0.26	0.21	ns	0.89	0.28	***
LOC115711337	CsSCL3	Signaling	0.00	0.13	ns	0.18	0.16	ns
LOC115702835	CsGID1B	Signaling	-0.04	0.21	ns	-1.67	0.47	***
LOC115719219	CsGID1C	Signaling	-0.14	0.14	ns	-0.42	0.18	*
LOC115712738	CsSLY1	Signaling	-0.40	0.17	*	-0.27	0.16	ns
LOC115698964	CsSLY2	Signaling	0.08	0.31	ns	-0.04	0.26	ns
LOC115701130	CsSPY	Signaling	-0.06	0.09	ns	0.01	0.10	ns
LOC115698303	CsGASA1	GASA	-4.04	0.74	***	-0.33	0.51	ns
LOC115701397	CsGASA4	GASA	0.17	0.24	ns	1.33	0.43	***
LOC115721929	CsGASA5	GASA	-0.01	0.34	ns	0.07	0.36	ns
LOC115708697	CsGASA6	GASA	0.72	0.84	*	0.22	0.41	ns
LOC115710205	CsGASA6-like	GASA	0.78	1.01	ns	0.53	0.77	ns
LOC115715027	CsGASA7	GASA	0.00	0.60	ns	0.01	0.45	ns
LOC115703025	CsGASA8	GASA	0.05	0.38	ns	1.73	1.74	**
LOC115703246	CsGASA9	GASA	0.05	0.24	ns	0.15	0.30	ns
LOC115714466	CsGASA10	GASA	0.04	0.26	ns	-0.04	0.26	ns
LOC115698285	CsGASA11	GASA	-0.17	0.34	ns	0.13	0.30	ns
LOC133030008	CsGASA14	GASA	-0.03	0.25	ns	0.08	0.26	ns

Supplementary Table S3. Differential expression of gibberellin (GA) pathway genes at day 14 under IMF vs. FF and MF vs. IFF treatment contrasts. Log2 fold change (log2FC), standard error (lfcSE), and Benjamini-Hochberg adjusted p-value (padj) are shown for each gene from the candidate GA pathway gene list (Supplementary Table S1) that was detected in the floral-tissue RNA-seq dataset at day 14. Values reflect the overall treatment effect for each contrast, estimated independently of cultivar. Log2FC values were obtained using ashr shrinkage estimation (lfcShrink, DESeq2). Genes are grouped by functional category. padj are indicated as: ns, not significant (padj ≥ 0.05); *, padj < 0.05; **, padj < 0.01; ***, padj < 0.001.

LOCID	Name	Function	IMF v. FF log2FC	IMF v. FF lfcSE	IMF v. FF padj	MF v. IFF log2FC	MF v. IFF lfcSE	MF v. IFF padj
LOC115703738	CsGA1	Biosynthesis	0.55	0.25	**	2.33	0.23	***
LOC115712980	CsGA2	Biosynthesis	-0.54	0.08	***	0.19	0.08	*
LOC115695692	CsGA3	Biosynthesis	0.33	0.12	**	0.98	0.12	***
LOC115710887	CsKAO1	Biosynthesis	-1.43	0.21	***	0.76	0.19	***
LOC115715978	CsKAO2	Biosynthesis	-1.62	0.38	***	0.68	0.31	*
LOC115715725	CsGA20ox2	Precursor	0.08	0.17	ns	2.25	0.20	***
LOC115716895	CsGA20ox2-like	Precursor	0.63	0.71	ns	3.20	0.67	***
LOC115704439	CsGA20ox1D	Precursor	2.87	0.49	***	2.40	0.46	***
LOC115712114	CsGA20ox1B	Precursor	0.95	0.12	***	0.75	0.12	***
LOC115694716	CsGA20ox1D-like	Precursor	4.64	0.66	***	4.02	0.62	***
LOC115703660	CsGA20ox-like2	Precursor	6.66	0.76	***	4.43	0.59	***
LOC115705300	CsGA3ox1	Activation	0.49	0.20	**	-0.65	0.20	**
LOC115704454	CsGA3ox3A	Activation	0.18	0.47	ns	5.61	0.89	***
LOC115708157	CsCYP72A219A	Diversion	0.25	0.25	ns	-0.57	0.29	*
LOC115706712	CsCYP72A219B	Diversion	0.18	0.08	*	0.14	0.08	ns
LOC115707772	CsCYP72A219C	Diversion	-0.18	0.12	ns	-0.03	0.12	ns
LOC115706721	CsCYP72A219D	Diversion	0.44	0.35	ns	-0.70	0.36	*
LOC115706085	CsCYP714A1	Inactivation	-2.19	0.13	***	-0.83	0.13	***
LOC115706670	CsGA2ox1	Inactivation	-0.39	0.16	*	0.89	0.17	***
LOC115696581	CsGA2ox2	Inactivation	2.09	0.28	***	0.69	0.26	**
LOC115723423	CsGA2ox4	Inactivation	0.29	0.27	ns	-1.11	0.34	***
LOC115704443	CsGA2ox6A	Inactivation	0.36	0.22	ns	0.64	0.23	**
LOC133032535	CsGA2ox6B	Inactivation	0.05	0.40	ns	0.48	0.39	ns
LOC115701137	CsGA2ox7	Inactivation	0.51	0.28	*	-0.60	0.28	*
LOC115704748	CsGA2ox8A	Inactivation	0.05	0.25	ns	0.84	0.28	**
LOC115695970	CsGA2ox8B	Inactivation	0.14	0.31	ns	0.07	0.36	ns
LOC115710658	CsGA2ox9	Inactivation	0.09	0.11	ns	-1.35	0.13	***
LOC115712712	CsGAI	Signaling	-0.11	0.08	ns	0.16	0.08	ns
LOC115706185	CsSLR1	Signaling	-0.17	0.07	*	-0.64	0.07	***
LOC115711337	CsSCL3	Signaling	0.48	0.14	***	0.36	0.14	*
LOC115702835	CsGID1B	Signaling	0.37	0.13	**	-1.27	0.13	***
LOC115719219	CsGID1C	Signaling	0.01	0.09	ns	-0.08	0.09	ns
LOC115712738	CsSLY1	Signaling	0.09	0.09	ns	0.01	0.09	ns
LOC115698964	CsSLY2	Signaling	-0.72	0.21	***	-2.27	0.22	***
LOC115701130	CsSPY	Signaling	0.02	0.07	ns	-0.51	0.07	***
LOC115698303	CsGASA1	GASA	-0.86	0.36	**	1.42	0.35	***
LOC115701397	CsGASA4	GASA	-0.67	0.12	***	-0.95	0.12	***

LOCID	Name	Function	IMF v. FF log2FC	IMF v. FF lfcSE	IMF v. FF padj	MF v. IFF log2FC	MF v. IFF lfcSE	MF v. IFF padj
LOC115721929	CsGASA5	GASA	-1.35	0.35	***	4.08	0.30	***
LOC115708697	CsGASA6	GASA	0.09	0.14	ns	1.48	0.15	***
LOC115710205	CsGASA6-like	GASA	0.11	0.15	ns	0.15	0.16	ns
LOC115715027	CsGASA7	GASA	6.40	0.96	***	2.05	0.85	**
LOC115703025	CsGASA8	GASA	-0.75	0.16	***	-0.52	0.16	***
LOC115703246	CsGASA9	GASA	-0.20	0.14	ns	0.24	0.15	ns
LOC115714466	CsGASA10	GASA	0.47	0.28	*	2.38	0.30	***
LOC115698285	CsGASA11	GASA	-1.13	0.48	**	2.39	0.43	***
LOC133030008	CsGASA14	GASA	-0.68	0.21	***	-1.96	0.21	***

Supplementary Table S4. Total number of significantly differentially expressed genes (DEGs) identified by DESeq2 for each pairwise treatment comparison, in flower and leaf tissue, along with the number of genes upregulated and downregulated in each contrast. DEGs were defined as genes with an adjusted p-value < 0.05 and an absolute log2 fold change > 1. Upregulated and downregulated gene counts refer to the direction of change in the first-named treatment relative to the second.

Tissue	Comparison	Total_DEGs	Upregulated	Downregulated
Flower (day 14)	IMF vs FF	3245	2553	692
Flower (day 14)	MF vs FF	7642	4965	2677
Flower (day 14)	MF vs IFF	5631	3825	1806
Leaf (day 1)	IMF vs FF	1133	884	249
Leaf (day 1)	MF vs FF	3345	2063	1282
Leaf (day 1)	MF vs IFF	917	473	444

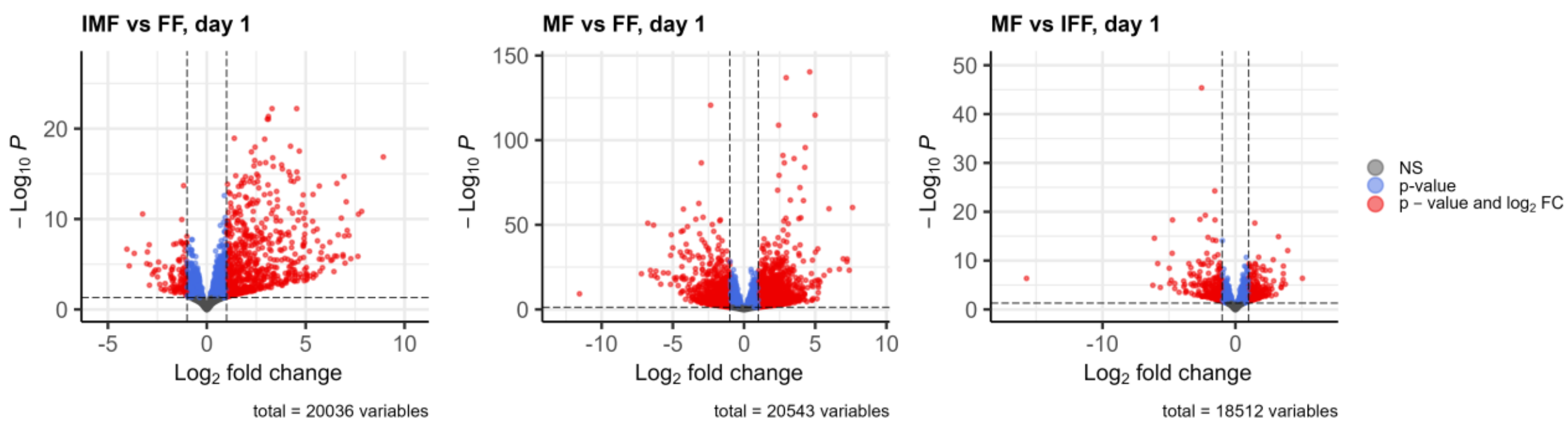

Supplementary Figure S1. Volcano plots of differentially expressed genes in leaf tissue at day 1, across three treatment contrasts. Volcano plots showing log2 fold change (x-axis) versus -log10 adjusted p-value (y-axis) for genes tested in each pairwise comparison: IMF vs. FF, MF vs. FF, and MF vs. IFF. Each point represents a single gene; genes are colored according to the fold change or significance threshold. The total number of genes tested (those with a non-missing adjusted p-value following DESeq2's independent filtering) is indicated below each panel.

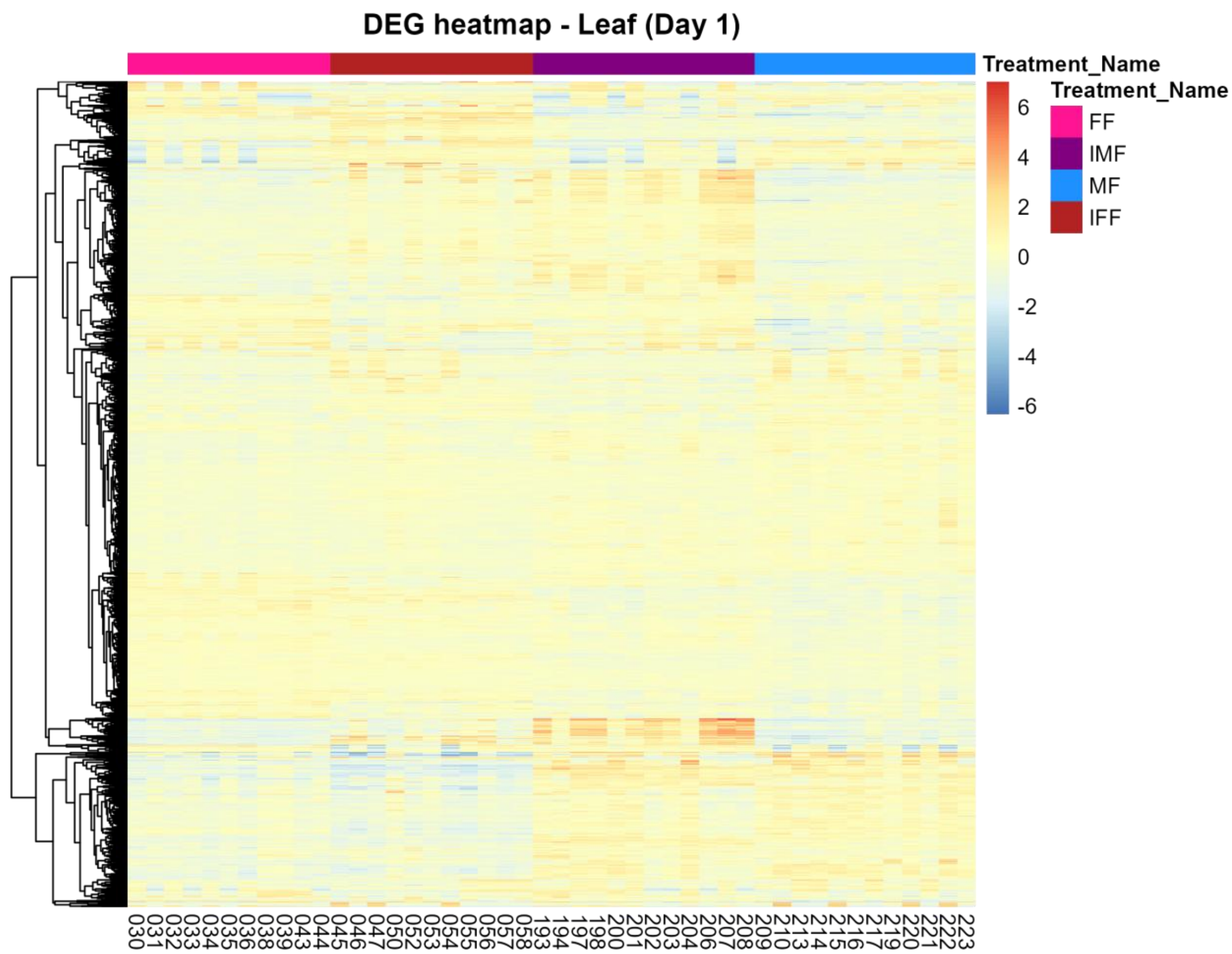

Supplementary Figure S2. Heatmap of differentially expressed genes in leaf tissue at day 1, across treatment groups. Heatmap showing variance-stabilizing-transformed (vst), row-centered expression values for the union of 4632 significantly differentially expressed genes (padj < 0.05, |log2FC| > 1) identified across the three treatment contrasts of interest (IMF vs. FF, MF vs. FF, and MF vs. IFF). Each row represents a gene and each column represents an individual sample; color indicates expression level relative to that gene's mean across samples. Samples are grouped by treatment.

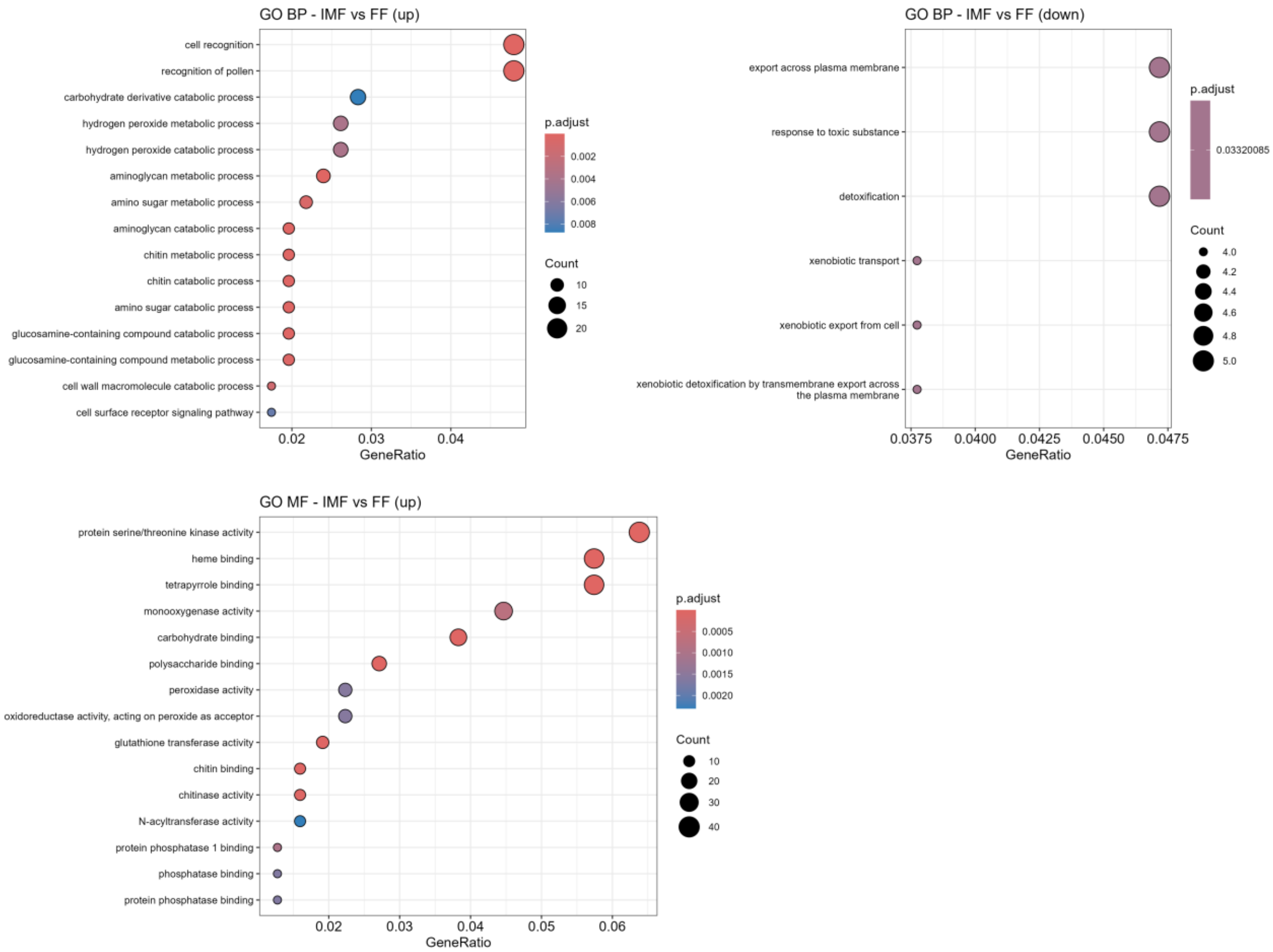

Supplementary Figure S3. Gene Ontology (GO) enrichment analysis of differentially expressed genes in leaf tissue, IMF vs. FF contrast, at day 1. Dot plots showing enriched GO terms among genes significantly upregulated (left column) and downregulated (right column) in the IMF vs. FF comparison, for the Biological Process (BP, top row) and Molecular Function (MF, bottom row) ontologies. For each panel, the x-axis (GeneRatio) indicates the proportion of input DEGs annotated to a given GO term relative to the total number of annotated DEGs; dot size reflects the number of genes (Count) contributing to each term, and dot color indicates the adjusted p-value (p.adjust), with red colors indicating stronger enrichment. Only GO terms with adjusted p-value < 0.05 are shown. Note that no GO Molecular Function terms were significantly enriched among downregulated genes in this contrast.

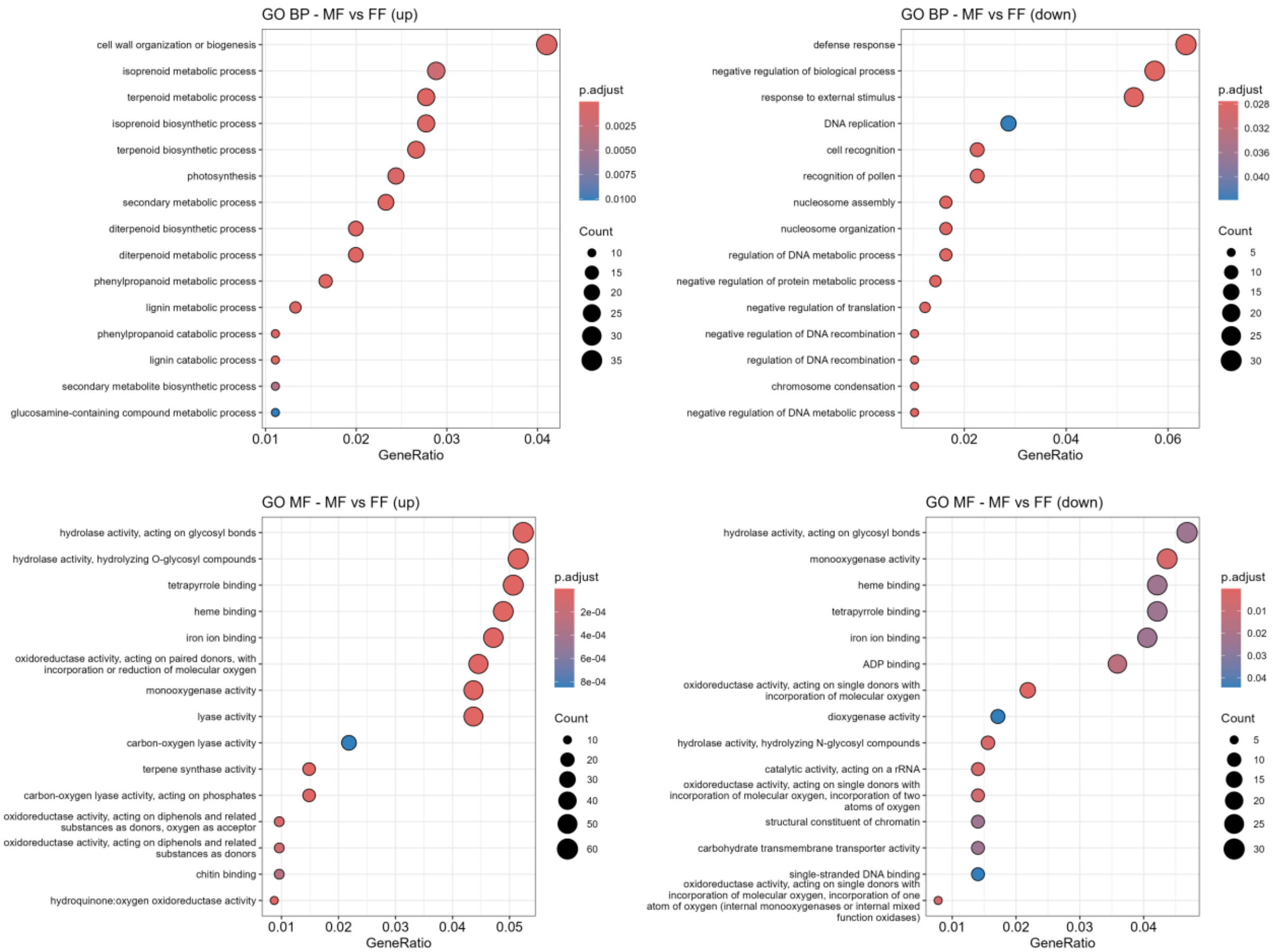

Supplementary Figure S4. Gene Ontology (GO) enrichment analysis of differentially expressed genes in leaf tissue, MF vs. FF contrast, at day 1. Dot plots showing enriched GO terms among genes significantly upregulated (left column) and downregulated (right column) in the MF vs. FF comparison, for the Biological Process (BP, top row) and Molecular Function (MF, bottom row) ontologies. For each panel, the x-axis (GeneRatio) indicates the proportion of input DEGs annotated to a given GO term relative to the total number of annotated DEGs; dot size reflects the number of genes (Count) contributing to each term, and dot color indicates the adjusted p-value (p.adjust), with red colors indicating stronger enrichment. Only GO terms with adjusted p-value < 0.05 are shown.

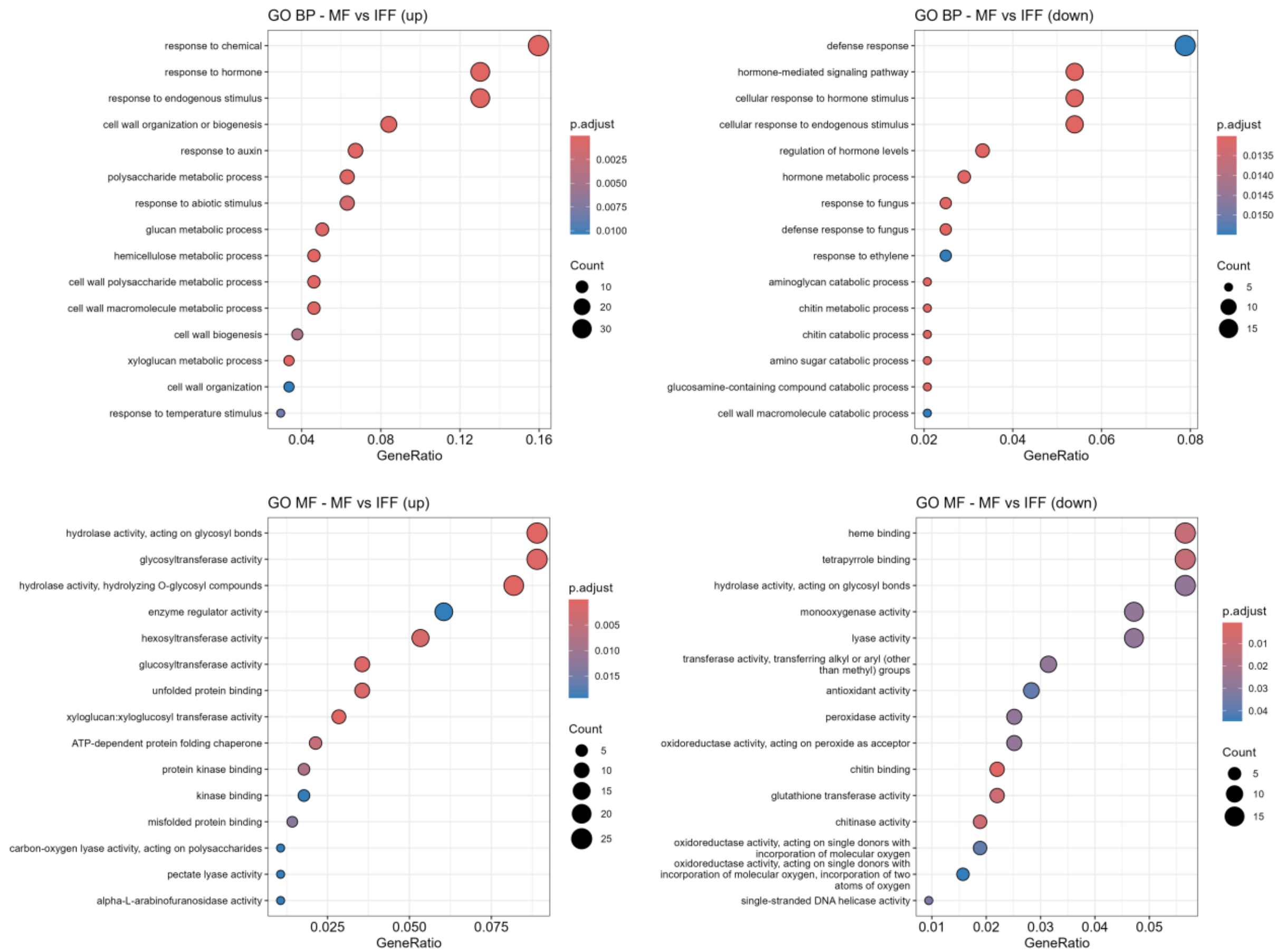

Supplementary Figure S5. Gene Ontology (GO) enrichment analysis of differentially expressed genes in leaf tissue, MF vs. IFF contrast, at day 1. Dot plots showing enriched GO terms among genes significantly upregulated (left column) and downregulated (right column) in the MF vs. IFF comparison, for the Biological Process (BP, top row) and Molecular Function (MF, bottom row) ontologies. For each panel, the x-axis (GeneRatio) indicates the proportion of input DEGs annotated to a given GO term relative to the total number of annotated DEGs; dot size reflects the number of genes (Count) contributing to each term, and dot color indicates the adjusted p-value (p.adjust), with red colors indicating stronger enrichment. Only GO terms with adjusted p-value < 0.05 are shown.

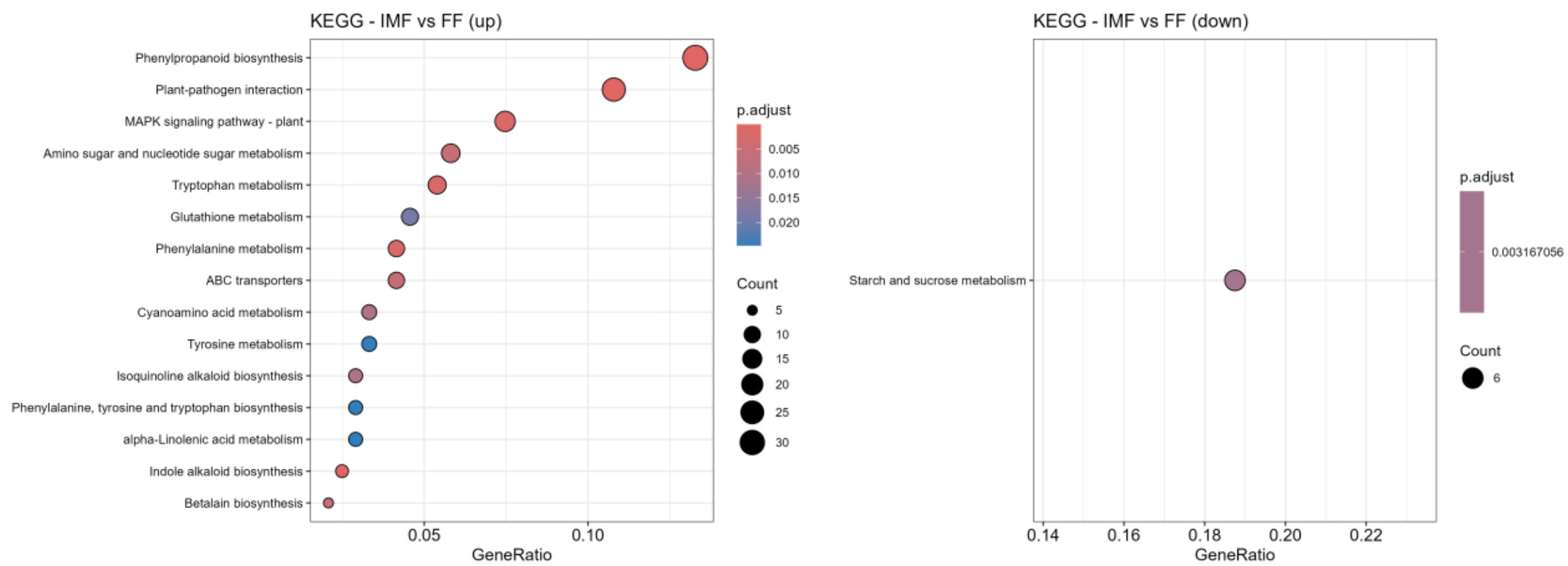

Supplementary Figure S6. KEGG pathway enrichment analysis of differentially expressed genes in leaf tissue, IMF vs. FF contrast, at day 1. Dot plots showing KEGG pathways enriched among genes significantly upregulated (left) and downregulated (right) in the IMF vs. FF comparison. The x-axis (GeneRatio) indicates the proportion of input DEGs annotated to a given pathway relative to the total number of DEGs with Entrez gene ID mappings; dot size reflects the number of genes (Count) contributing to each pathway, and dot color indicates the adjusted p-value (p.adjust), with red colors indicating stronger enrichment. Only pathways with adjusted p-value < 0.05 are shown. KEGG enrichment was performed using clusterProfiler::enrichKEGG() (organism code "csav") on genes mapped from gene symbol to Entrez ID via the *Cannabis sativa* AnnotationHub OrgDb (accession AH120088).

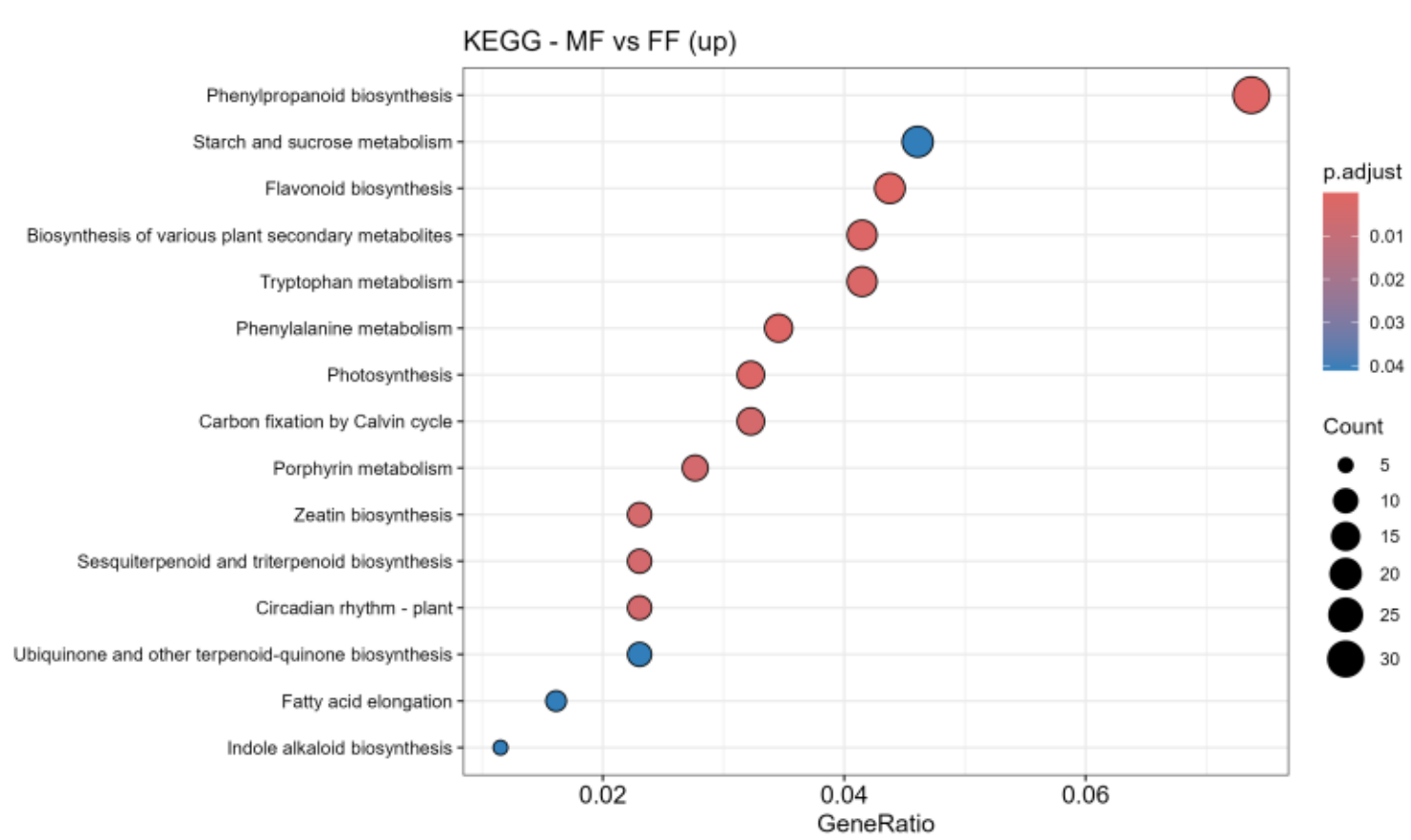

Supplementary Figure S7. KEGG pathway enrichment analysis of differentially expressed genes in leaf tissue, MF vs. FF contrast, at day 1. Dot plots showing KEGG pathways enriched among genes significantly upregulated in the MF vs. FF comparison. The x-axis (GeneRatio) indicates the proportion of input DEGs annotated to a given pathway relative to the total number of DEGs with Entrez gene ID mappings; dot size reflects the number of genes (Count) contributing to each pathway, and dot color indicates the adjusted p-value (p.adjust), with red colors indicating stronger enrichment. Only pathways with adjusted p-value < 0.05 are shown. KEGG enrichment was performed using clusterProfiler::enrichKEGG() (organism code "csav") on genes mapped from gene symbol to Entrez ID via the *Cannabis sativa* AnnotationHub OrgDb (accession AH120088). Note that no KEGG pathway enrichment terms were significantly enriched among downregulated genes in this contrast.

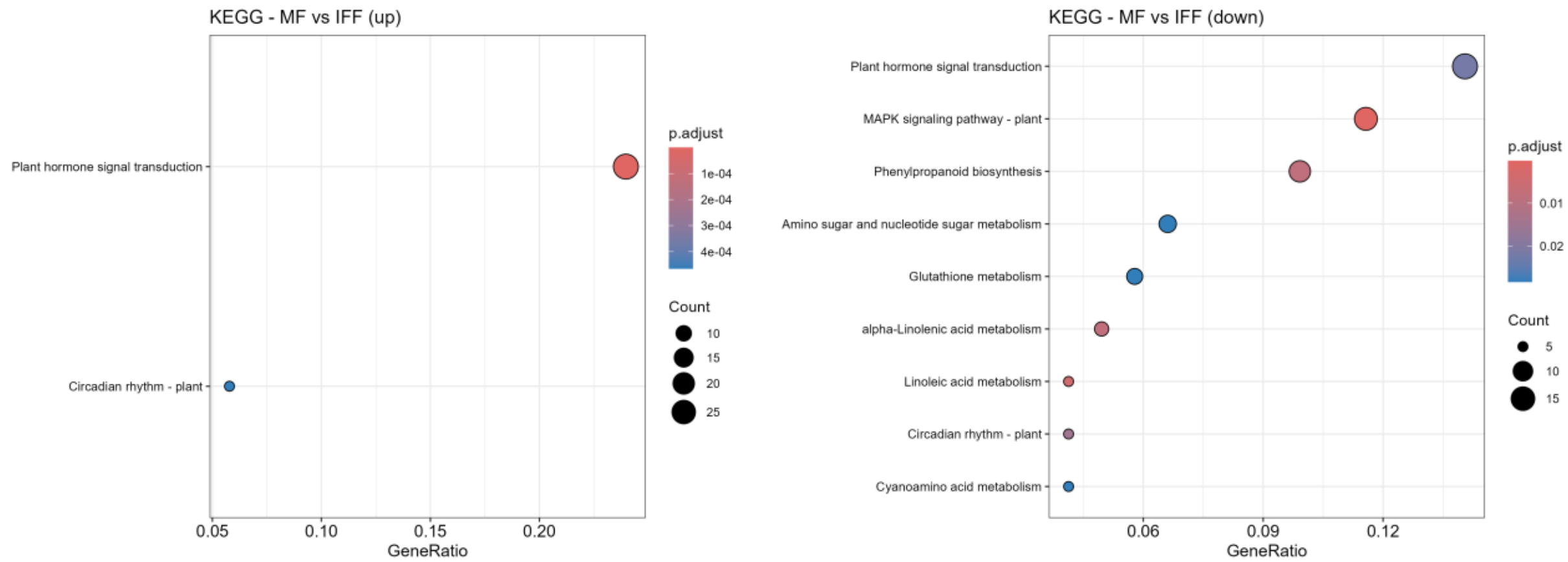

Supplementary Figure S8. KEGG pathway enrichment analysis of differentially expressed genes in leaf tissue, MF vs. IFF contrast, at day 1. Dot plots showing KEGG pathways enriched among genes significantly upregulated in the MF vs. IFF comparison. The x-axis (GeneRatio) indicates the proportion of input DEGs annotated to a given pathway relative to the total number of DEGs with Entrez gene ID mappings; dot size reflects the number of genes (Count) contributing to each pathway, and dot color indicates the adjusted p-value (p.adjust), with red colors indicating stronger enrichment. Only pathways with adjusted p-value < 0.05 are shown. KEGG enrichment was performed using clusterProfiler::enrichKEGG() (organism code "csav") on genes mapped from gene symbol to Entrez ID via the *Cannabis sativa* AnnotationHub OrgDb (accession AH120088).

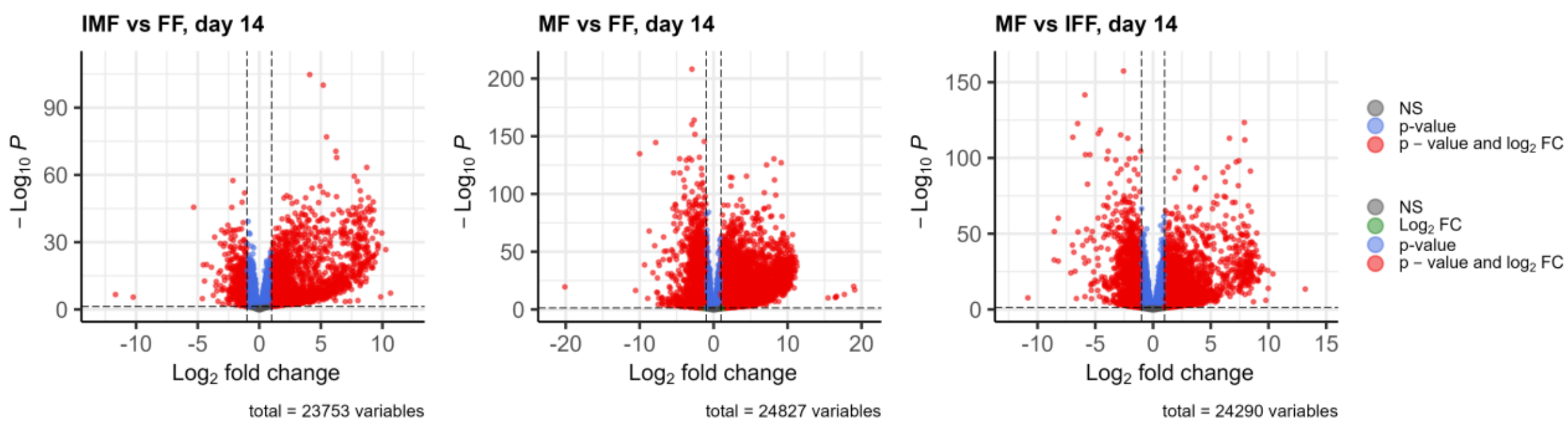

Supplementary Figure S9. Volcano plots of differentially expressed genes in floral tissue at day 14, across three treatment contrasts. Volcano plots showing log2 fold change (x-axis) versus -log10 adjusted p-value (y-axis) for genes tested in each pairwise comparison: IMF vs. FF, MF vs. FF, and MF vs. IFF. Each point represents a single gene; genes are colored according to the fold change or significance threshold. The total number of genes tested (those with a non-missing adjusted p-value following DESeq2's independent filtering) is indicated below each panel.

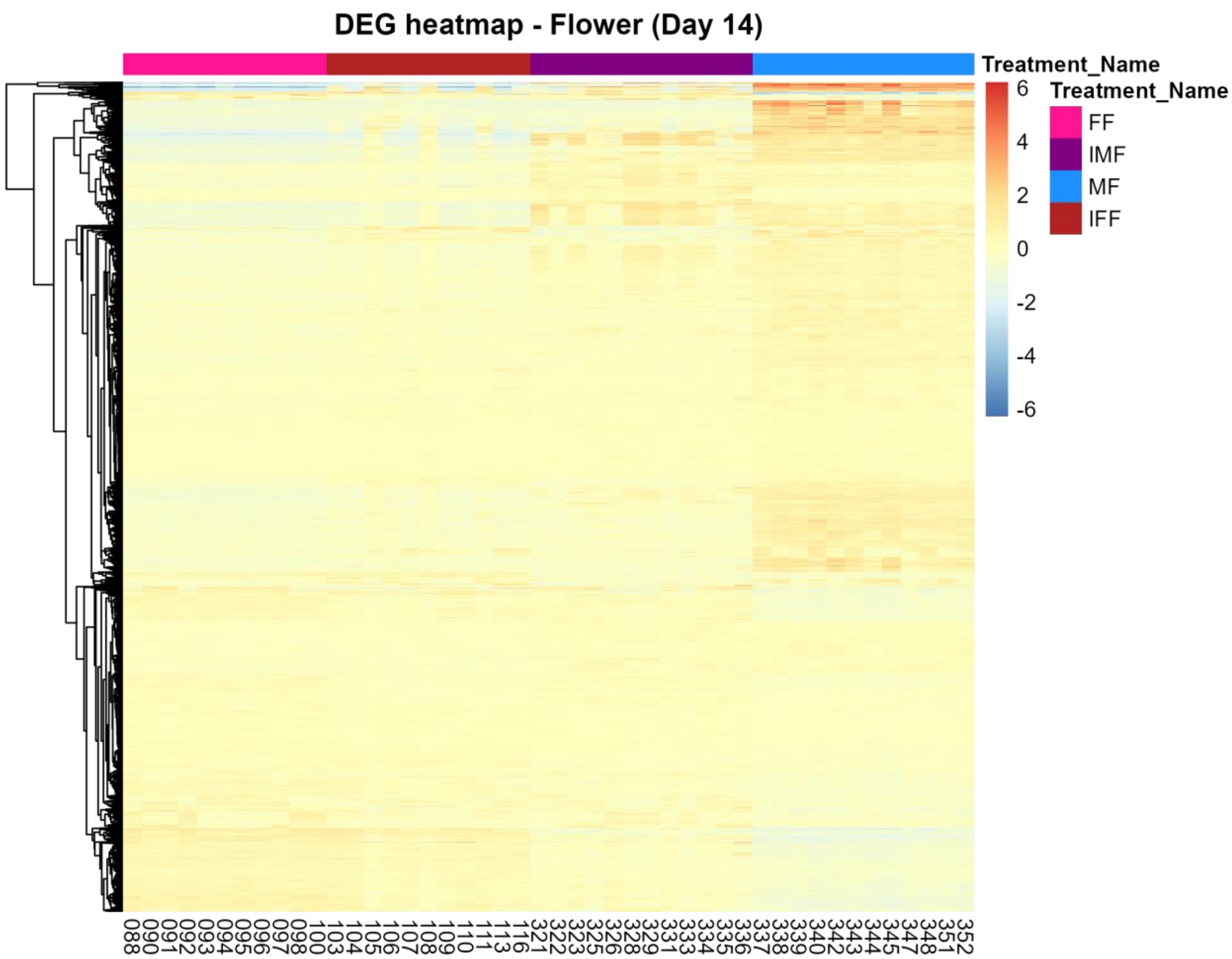

Supplementary Figure S10. Heatmap of differentially expressed genes in floral tissue at day 14, across treatment groups. Heatmap showing variance-stabilizing-transformed (vst), row-centered expression values for the union of 8603 significantly differentially expressed genes (padj < 0.05, |log2FC| > 1) identified across the three treatment contrasts of interest (IMF vs. FF, MF vs. FF, and MF vs. IFF). Each row represents a gene and each column represents an individual sample; color indicates expression level relative to that gene's mean across samples. Samples are grouped by treatment.

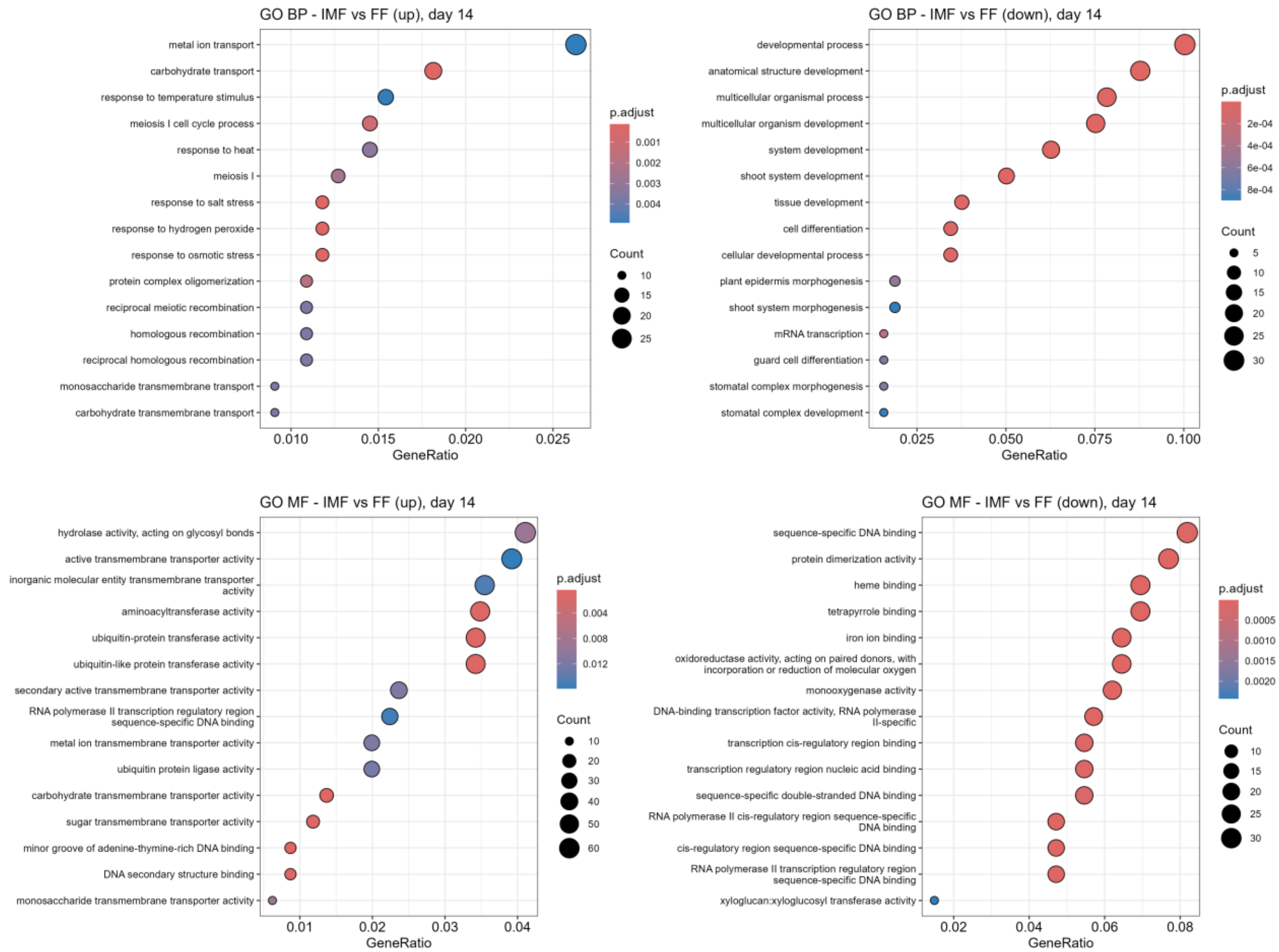

Supplementary Figure S11. Gene Ontology (GO) enrichment analysis of differentially expressed genes in floral tissue, IMF vs. FF contrast, at day 14. Dot plots showing enriched GO terms among genes significantly upregulated (left column) and downregulated (right column) in the IMF vs. FF comparison, for the Biological Process (BP, top row) and Molecular Function (MF, bottom row) ontologies. For each panel, the x-axis (GeneRatio) indicates the proportion of input DEGs annotated to a given GO term relative to the total number of annotated DEGs; dot size reflects the number of genes (Count) contributing to each term, and dot color indicates the adjusted p-value (p.adjust), with red colors indicating stronger enrichment. Only GO terms with adjusted p-value < 0.05 are shown. Note that no GO Molecular Function terms were significantly enriched among downregulated genes in this contrast.

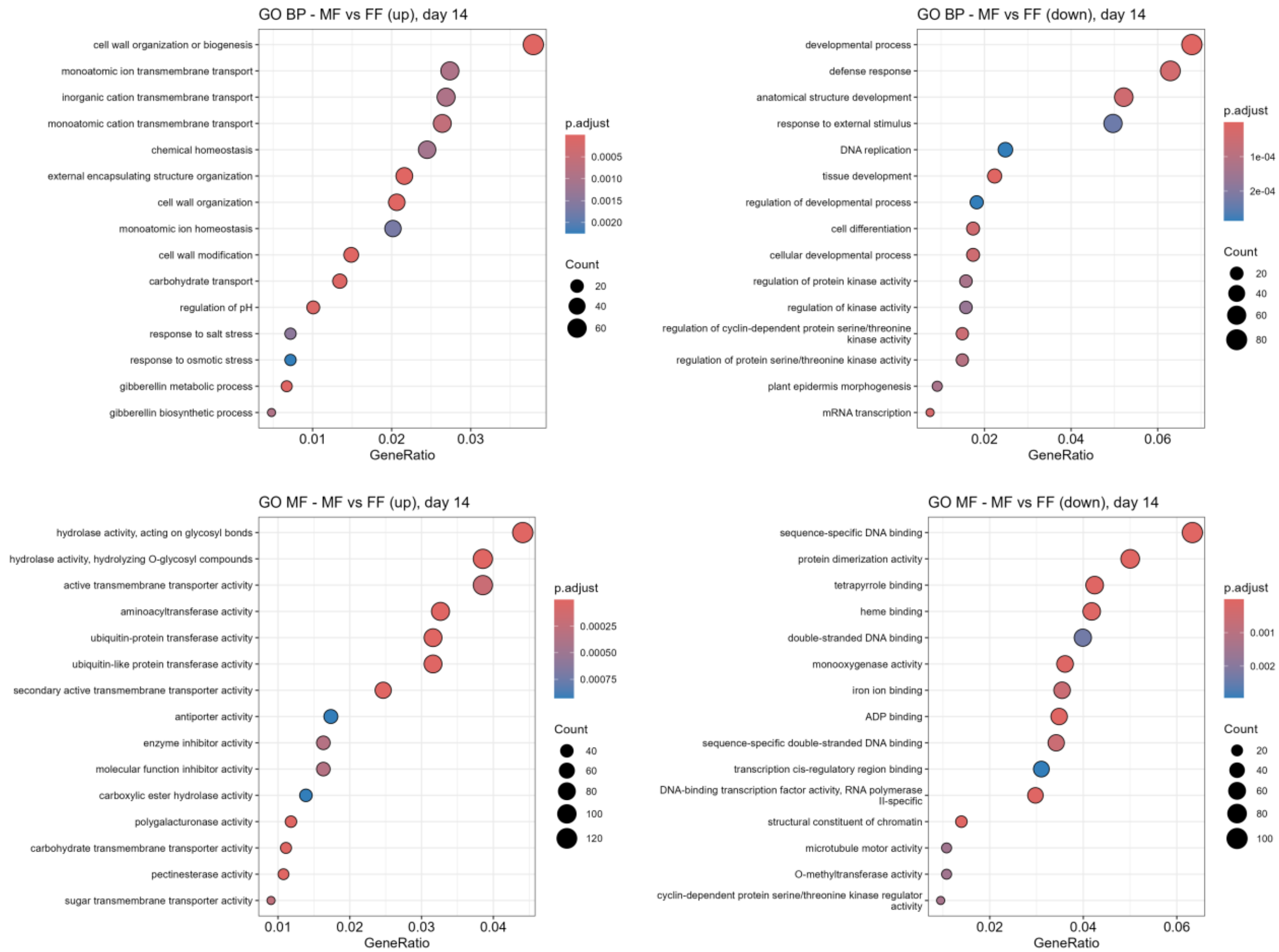

Supplementary Figure S12. Gene Ontology (GO) enrichment analysis of differentially expressed genes in floral tissue, MF vs. FF contrast, at day 14. Dot plots showing enriched GO terms among genes significantly upregulated (left column) and downregulated (right column) in the MF vs. FF comparison, for the Biological Process (BP, top row) and Molecular Function (MF, bottom row) ontologies. For each panel, the x-axis (GeneRatio) indicates the proportion of input DEGs annotated to a given GO term relative to the total number of annotated DEGs; dot size reflects the number of genes (Count) contributing to each term, and dot color indicates the adjusted p-value (p.adjust), with red colors indicating stronger enrichment. Only GO terms with adjusted p-value < 0.05 are shown.

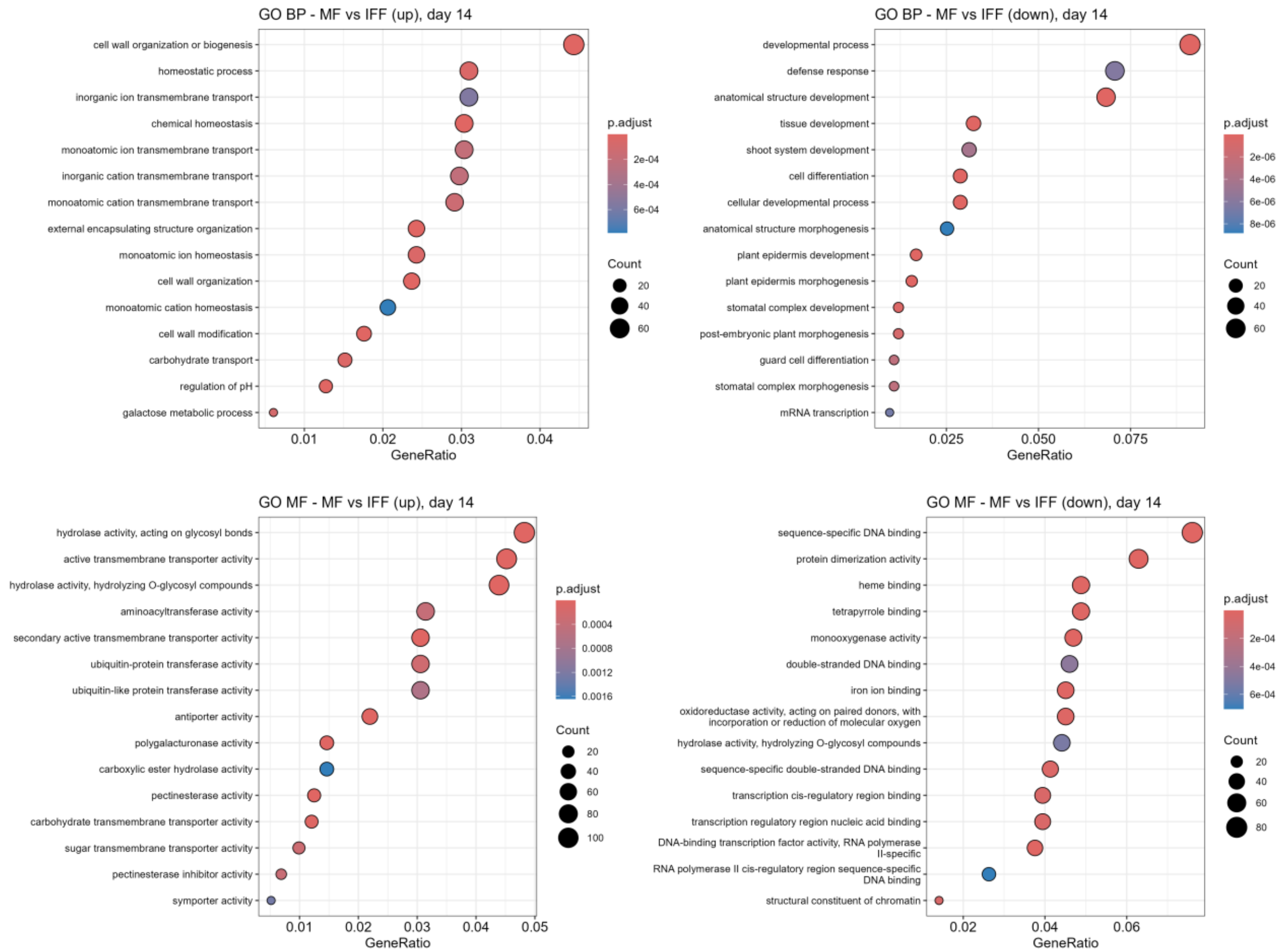

Supplementary Figure S13. Gene Ontology (GO) enrichment analysis of differentially expressed genes in floral tissue, MF vs. IFF contrast, at day 14. Dot plots showing enriched GO terms among genes significantly upregulated (left column) and downregulated (right column) in the MF vs. IFF comparison, for the Biological Process (BP, top row) and Molecular Function (MF, bottom row) ontologies. For each panel, the x-axis (GeneRatio) indicates the proportion of input DEGs annotated to a given GO term relative to the total number of annotated DEGs; dot size reflects the number of genes (Count) contributing to each term, and dot color indicates the adjusted p-value (p.adjust), with red colors indicating stronger enrichment. Only GO terms with adjusted p-value < 0.05 are shown.

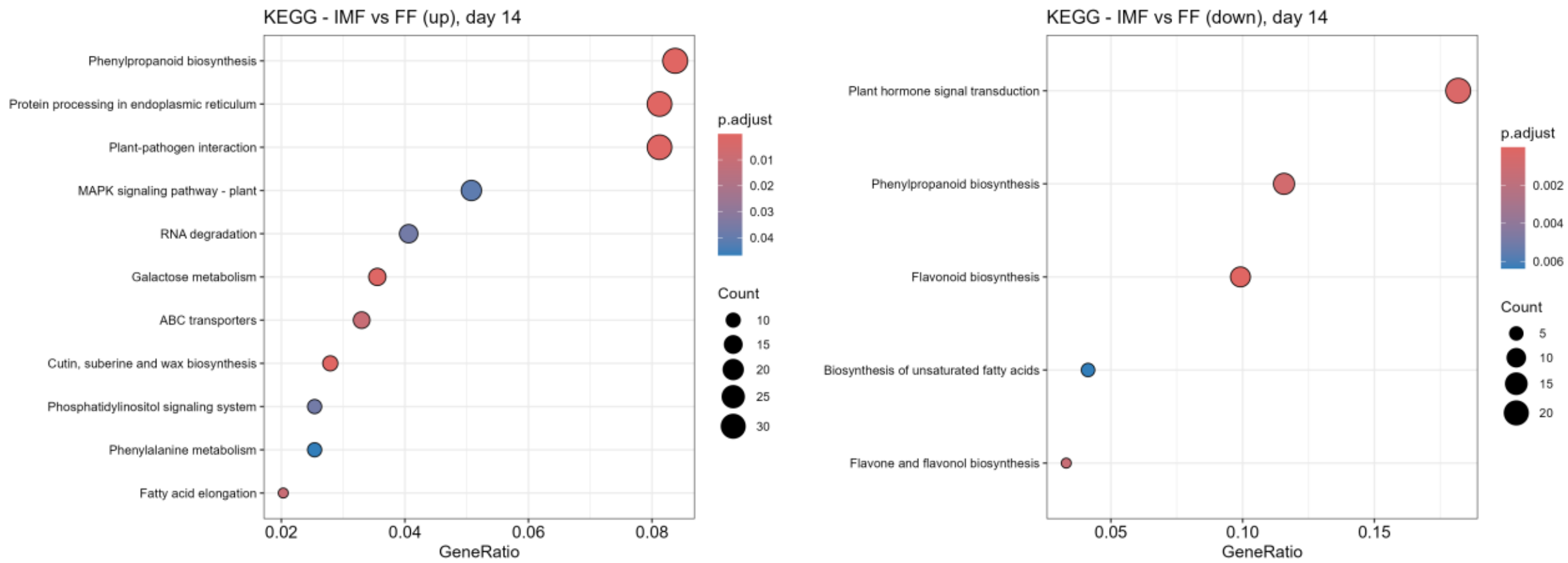

Supplementary Figure S14. KEGG pathway enrichment analysis of differentially expressed genes in floral tissue, IMF vs. FF contrast, at day 14. Dot plots showing KEGG pathways enriched among genes significantly upregulated in the IMF vs. FF comparison. The x-axis (GeneRatio) indicates the proportion of input DEGs annotated to a given pathway relative to the total number of DEGs with Entrez gene ID mappings; dot size reflects the number of genes (Count) contributing to each pathway, and dot color indicates the adjusted p-value (p.adjust), with red colors indicating stronger enrichment. Only pathways with adjusted p-value < 0.05 are shown. KEGG enrichment was performed using clusterProfiler::enrichKEGG() (organism code "csav") on genes mapped from gene symbol to Entrez ID via the *Cannabis sativa* AnnotationHub OrgDb (accession AH120088).

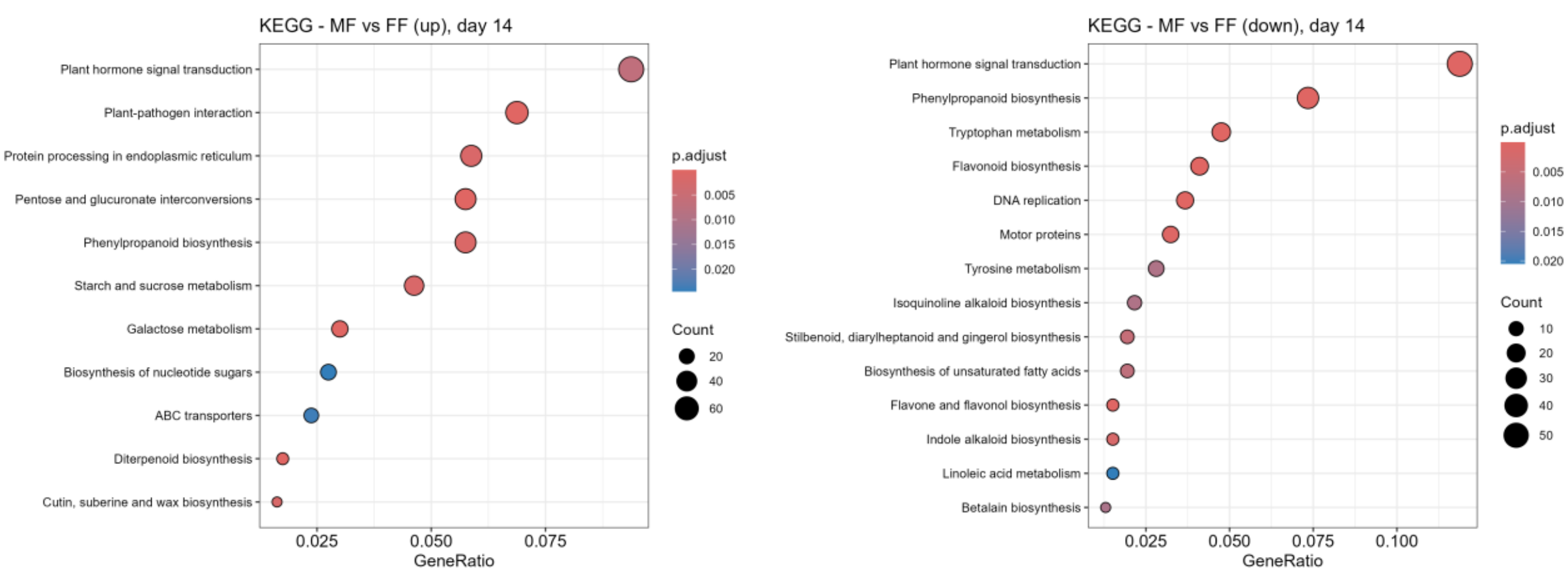

Supplementary Figure S15. KEGG pathway enrichment analysis of differentially expressed genes in floral tissue, MF vs. FF contrast, at day 14. Dot plots showing KEGG pathways enriched among genes significantly upregulated in the MF vs. FF comparison. The x-axis (GeneRatio) indicates the proportion of input DEGs annotated to a given pathway relative to the total number of DEGs with Entrez gene ID mappings; dot size reflects the number of genes (Count) contributing to each pathway, and dot color indicates the adjusted p-value (p.adjust), with red colors indicating stronger enrichment. Only pathways with adjusted p-value < 0.05 are shown. KEGG enrichment was performed using clusterProfiler::enrichKEGG() (organism code "csav") on genes mapped from gene symbol to Entrez ID via the *Cannabis sativa* AnnotationHub OrgDb (accession AH120088).

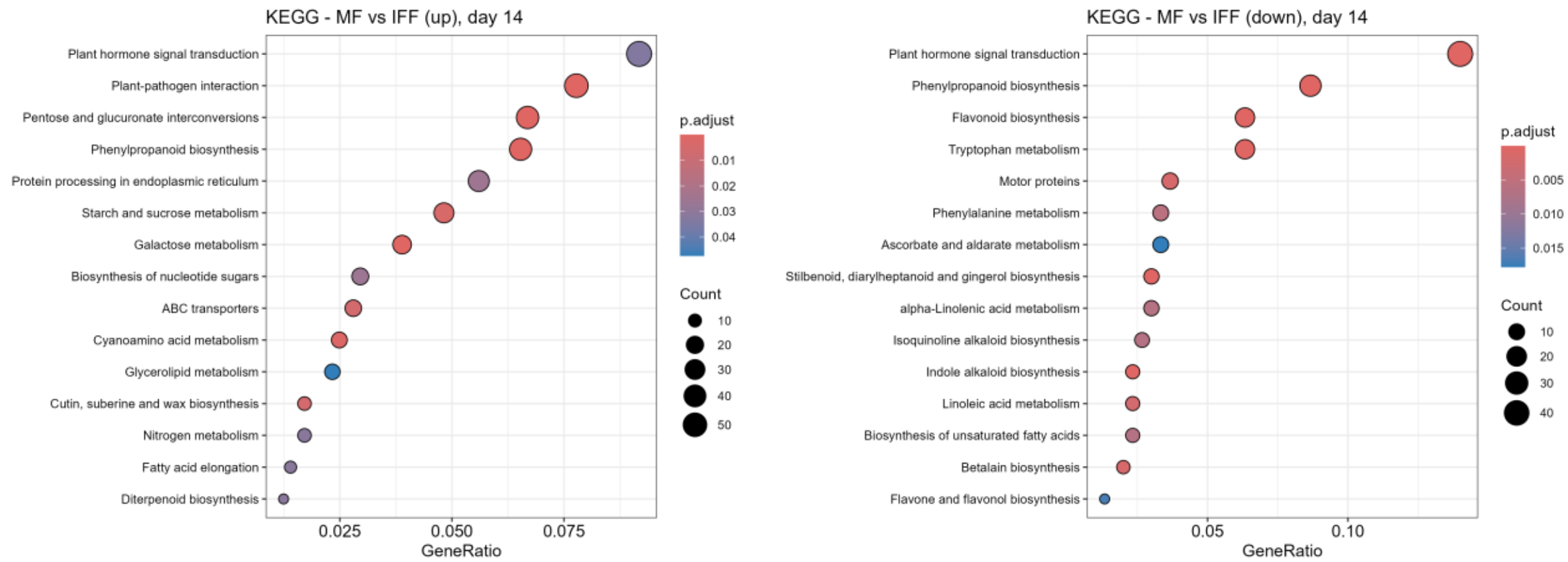

Supplementary Figure S16. KEGG pathway enrichment analysis of differentially expressed genes in floral tissue, MF vs. IFF contrast, at day 14. Dot plots showing KEGG pathways enriched among genes significantly upregulated (left) and downregulated (right) in the MF vs. IFF comparison. The x-axis (GeneRatio) indicates the proportion of input DEGs annotated to a given pathway relative to the total number of DEGs with Entrez gene ID mappings; dot size reflects the number of genes (Count) contributing to each pathway, and dot color indicates the adjusted p-value (p.adjust), with red colors indicating stronger enrichment. Only pathways with adjusted p-value < 0.05 are shown. KEGG enrichment was performed using clusterProfiler::enrichKEGG() (organism code "csav") on genes mapped from gene symbol to Entrez ID via the *Cannabis sativa* AnnotationHub OrgDb (accession AH120088).
